# Long-term disinfection of 3D-printed denture resin: physical and biological in vitro assessments

**DOI:** 10.1007/s10856-026-07049-8

**Published:** 2026-04-24

**Authors:** Amanda C. Ferro, Caroline C. de Oliveira, Bárbara L. Morais, Jonatas S. de Oliveira, Rodolfo D. Piazza, Rodrigo F. C. Marques, Carlos Mota, Matthew B. Baker, Janaina H. Jorge

**Affiliations:** 1 Department of Dental Materials and Prosthodontics, School of Dentistry, São Paulo State University (UNESP), Araraquara, Brazil; 2 Department of Complex Tissue Regeneration, Institute for Technology-Inspired Regenerative Medicine (MERLN), Maastricht University, Maastricht, The Netherlands; 3 Department of Morphology, Genetics, Orthodontics and Pediatric Dentistry, School of Dentistry, São Paulo State University (UNESP), Araraquara, Brazil; 4 Department of Analytical, Physico-chemistry and Inorganic Chemistry, Institute of Chemistry, São Paulo State University (UNESP), Araraquara, Brazil; 5 Department of Instructive Biomaterials Engineering, Institute for Technology-Inspired Regenerative Medicine (MERLN), Maastricht University, Maastricht, The Netherlands

## Abstract

This study evaluated the effects of prolonged overnight immersion in disinfectant solutions on the physical and biological properties of 3D-printed and heat-polymerized polymethyl methacrylate (PMMA) denture base materials. Four solutions were tested: distilled water (control), 1% sodium hypochlorite, 2% chlorhexidine digluconate, and a disinfectant soap (Lifebuoy®). Daily cycles of 8 h in disinfectant solutions and 16 h in distilled water were performed for up to 6 months to represent overnight disinfection and daily use. The evaluated parameters included color change, water contact angle, Vickers hardness, surface roughness and topography, residual antimicrobial activity against *Candida albicans* biofilm, and cytotoxicity in L-929 cells. Color change remained within clinically acceptable thresholds for all groups, with Lifebuoy® showing values comparable to the control. Water contact angles decreased after immersion, while surface roughness was stable up to 3 months and decreased at 6 months, particularly in PMMA. Hardness increased in heat-polymerized specimens, whereas 3D-printed materials showed greater stability over time. 3D-printed resins exhibited higher *C. albicans* biofilm formation than PMMA. Chlorhexidine digluconate resulted in the greatest reduction in fungal growth and metabolic activity, followed by sodium hypochlorite and Lifebuoy®. Most groups showed no cytotoxic effects, except for moderate cytotoxicity of chlorhexidine at 3 months. In conclusion, 3D-printed resin showed superior physical performance, while PMMA demonstrated lower *Candida* colonization. Chlorhexidine was the most effective antibiofilm agent despite time-dependent cytotoxicity, while Lifebuoy® served as a non-cytotoxic alternative.

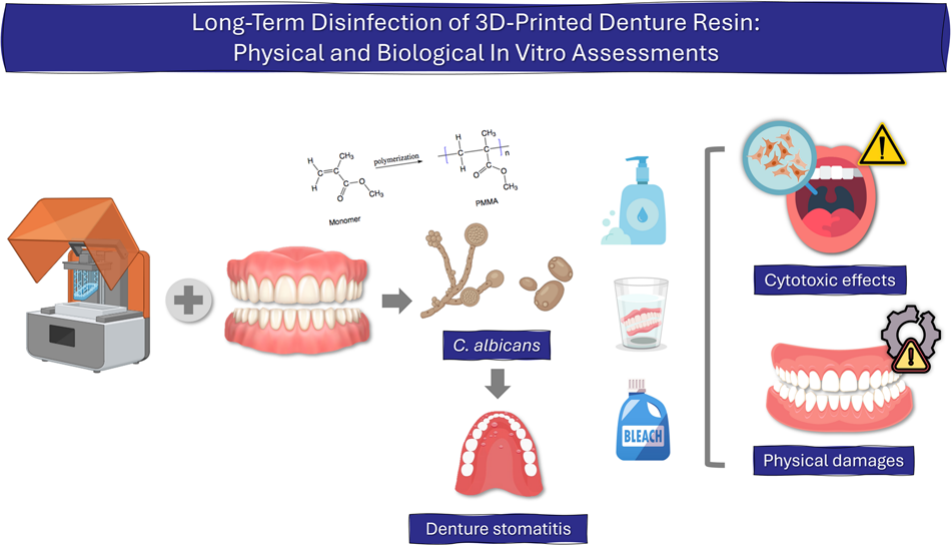

## Introduction

Traditionally, removable dentures are fabricated from heat-polymerized poly(methyl methacrylate) (PMMA). In recent years, advances in digital dentistry have enabled the adoption of additive manufacturing (AM) technologies, including three-dimensional (3D) printing, as part of digital workflow for dental applications. This approach has been increasingly incorporated into clinical practice due to advantages such as reduced chairside time and fewer clinical appointments [1, 2].

Within the AM framework, vat photopolymerization-based 3D printing operates by selectively polymerizing a liquid photopolymer resin using ultraviolet (UV) light, layer by layer, as the build platform moves to form the desired object [3]. Among these systems, digital light processing (DLP) printers are widely used in prosthodontics for denture fabrication. Previous studies have demonstrated that maxillary denture bases manufactured using DLP technology demonstrated improved trueness and tissue surface adaptation compared with liquid crystal display (LCD) printer and conventional methods [4, 5].

From a materials perspective, the polymers used in 3D printing for prosthodontic fabrication are relatively new and remain insufficiently investigated. Recent studies have reported increased microbial adhesion to 3D-printed materials and emphasized the limited data available regarding other properties, such as cytotoxicity associated with residual monomer release [6, 7], as well as their performance under routine denture maintenance protocols for denture stomatitis prevention.

Denture stomatitis (DS) affects more than 70% of complete denture wearers [8, 9]. Although its etiology is multifactorial, the adhesion and proliferation of *Candida albicans* biofilm on denture surfaces are directly associated with its development and progression [10]. This microorganism can also colonize other medical devices, including catheters, prostheses, and endotracheal tubes, where it forms pathogenic biofilms that may contribute to systemic infections, particularly in institutionalized individuals [11–13].

In this context, the intaglio surface of denture bases may serve as a reservoir for biofilm accumulation due to its irregularities and porosity [14, 15]. Mechanical cleansing, such as brushing with soap or toothpaste, is the most common method for biofilm control, although it may be insufficient, particularly among elderly individuals with reduced manual dexterity [16, 17]. Therefore, chemical disinfection is well established for conventional dentures, but evidence and clinical guidelines for 3D-printed materials are still lacking [18].

Among chemical cleansers, sodium hypochlorite and chlorhexidine digluconate are known due to their antifungal and antibiofilm activity [19, 20]. However, their prolonged use has been associated with adverse effects, including cytotoxicity, color alteration of the acrylic base, reduced flexural strength and metal corrosion [21–23]. Thus, neutral liquid soaps have emerged as an affordable and widely available alternative, with previous studies reporting antimicrobial efficacy without cytotoxic effects or changes to denture resin surfaces [24, 25]. Despite this, their long-term performance on 3D-printed resins remains unclear.

In clinical practice, dentures are usually soaked overnight in cleansing solutions over extended periods. Short-term laboratory protocols fail to replicate these conditions, reinforcing the need for more realistic immersion models. Moreover, chemical disinfectants do not come into direct contact with oral tissues; instead, residual agents absorbed and retained within the acrylic base after rinsing may represent the actual source of tissue exposure [26–28]. These residues may contribute to cytotoxic effects while simultaneously maintaining antimicrobial activity by preventing recolonization [28].

Considering these aspects and the emergence of new manufacturing technologies, this study evaluated the physical and biological responses of a 3D-printed denture base resin after long-term overnight immersion in disinfectant solutions, addressing changes in physical properties, microbial inhibition, and potential tissue exposure to residual agents to support clinical hygiene guidelines for 3D-printed dentures. The null hypothesis was that long-term overnight immersion in disinfectant solutions would not affect the physical and biological properties of the resins, and that no differences would be observed between the two denture fabrication methods (conventional and 3D printing).

## Methods

### Specimen fabrication

Disc-shaped specimens (14 mm diameter × 1.2 mm thickness) were prepared from both 3D printing denture base resin (Cosmos Denture, Yller Biomaterials, Brazil) and heat-polymerized PMMA (Vipi Wave, Vipi Dental Products, Brazil). Information regarding manufacturer-reported composition and processing conditions of the materials was provided in Table 1.

**Table 1:** Description of the 3D printing denture base resin and heat-polymerized PMMA used in this study

Resins	Color	Composition	Processing instructions according to manufacturers
Cosmos Denture Yller Biomaterials, Brazil	Pink	Methacrylate oligomer (>80%); Diphenyl-2,4,6-trimethylbenzoyl phosphine oxide (<5%); Phosphine oxide, phenylbis(2,4,6-trimethylbenzoyl) (<5%); Titanium dioxide (<1%).	Wash the objects in isopropyl alcohol or ethanol using two consecutive baths of 5 min each. Post-cure in a 72 W UV chamber for up to 10 min.
Vipi Wave VIPI Dental Materials, Brazil	Medium pink	Powder: Polymethylmethacrylate, Polypropylene, Pigments Liquid: Methyl methacrylate (>94%), EDMA (Crosslink), Inhibitor, Terpinolen.	Mix 6.5 mL of monomer with 14 g of polymer powder in a vessel, preferably glass, until the plastic phase is reached. Polymerize in a 500 W microwave oven for 20 min at 20–30% power, followed by 5 min at 80–100% power.

3D-printed specimens were designed in a CAD software (Meshmixer v.3.5.474, Autodesk, Inc.), exported as STL files, and sliced (FlashDLPrint v2.3.0). Printing was performed using a Flashforge Hunter printer (Zhejiang Flashforge 3D Technology Co., Ltd., China) at 90° orientation and 0.05 mm layer thickness, based on a previous study [29]. Post-processing included cleaning with isopropyl alcohol under agitation for 5 min and post-curing in a CiclOne unit (dOne 3D, Brazil), according to the manufacturer’s recommendations (Table 1).

Heat-polymerized PMMA specimens were prepared in metallic molds using conventional flasking technique. The powder/liquid ratio was manipulated, packed in the plastic stage, pressed hydraulically (9.8–14.7 N),and polymerized in a domestic microwave oven (500 W, Brastemp, Brazil) for 20 min at 20% power followed by 5 min at 80%, following manufacturer’s instructions (Table 1). After cooling, specimens were deflasked, excess material was removed with a Maxicut bur (Lesfils de August Malleifer, Switzerland), and ultrasonic cleaning was performed in distilled water for 10 min (see supplementary information, Fig. S1).

Prior to testing, surface roughness was standardized for all specimens to ensure comparable baseline conditions by removing printing lines from 3D-printed specimens and surface irregularities associated with the conventional fabrication. Accordingly, specimens were manually finished under continuous running water using 220-grit silicon carbide paper [30]. Finishing was performed by the same operator using uniform circular movements under light and constant pressure for approximately 30–60 s. Subsequently, all specimens were stored in distilled water at 37 °C for 48 h [31]. This procedure, consistent with routine clinical practice, was adopted to minimize the influence of residual monomers on the evaluations further performed.

### Experimental groups and immersion protocol

The specimens were allocated into the experimental groups described in Table 2. Four immersion solutions were selected, including commonly used agents for overnight denture disinfection and storage at their respective standard concentrations for complete dentures [22, 28, 32]: 1% sodium hypochlorite (SH), 2% chlorhexidine digluconate (CD), 0.78% Lifebuoy® soap solution (LS, corresponding to the minimum inhibitory concentration for *C. albicans* [24, 25]), and distilled water (DW) as the control group. Additionally, the pH of each solution was measured using a calibrated digital pH meter (K39-1420A; Kasvi) at 25 °C and the values were presented in Table 2.

**Table 2:** Description of the immersion solutions and overnight exposure periods

Representation	Solution	Immersion period	pH
DW	Distilled water (control group)	0, 1, 3, and 6 months	6.3
SH	1% sodium hypochlorite		11.8
CD	2% chlorhexidine digluconate		5.8
LS	0.78% Lifebuoy® soap solution		4.3

The immersion protocol consisted of daily cycles of 8 h in the tested solutions to simulate overnight denture disinfection, followed by 16 h in distilled water at 37 °C to simulate daily use. All solutions were renewed daily, and specimens were rinsed under running water between cycles.

The evaluated time points were T0 (baseline), T1 (1 month), T3 (3 months), and T6 (6 months). The baseline condition (T0) corresponded to a 48-h storage period in distilled water after specimen fabrication. At each endpoint, specimens were rinsed under running water prior to physical and biological testing to remove residual disinfectant solution.

### Physical evaluation

#### Color change (ΔE00)

To assess color stability, measurements were performed using the CIE L*a*b* color space, as defined by the Commission Internationale de L’Eclairage (CIE, 1978) [33]. Measurements were performed with specimens positioned against a black background to simulate the oral cavity, using a portable spectrophotometer (Color Guide 45/0, PCB 6807 BYK-Gardner GmbH, Germany) with a 4 mm aperture and daylight as illuminant (D65) [34]. To ensure consistency, all readings were taken under controlled lighting and with the device aligned to the center of each specimen. Color differences (ΔE00) were calculated using the CIEDE2000 formula (1), based on the CIELAB color space [35].

1 $$\begin{array}{l}\Delta E00=[{(\Delta {L}^{{\prime} }/{K}_{L}{S}_{L})}^{2}+{(\Delta {C}^{{\prime} }/{K}_{C}{S}_{C})}^{2}+{(\Delta {H}^{{\prime} }/{K}_{H}{S}_{H})}^{2}\\ \,\,\,\,\,\,\,\,\,\,+{R}_{T}(\Delta {C}^{{\prime} }/{K}_{C}{S}_{C})(\Delta {H}^{{\prime} }/{K}_{H}{S}_{H})]1/2\end{array}$$

For clinical interpretation, ΔE00 values were classified as follows: ≤1.7, imperceptible; 1.7–4.1, clinically acceptable; ≥4.1, clinically unacceptable, based on Ren et al. [36] classification. To account for data variability, a conservative criterion was adopted by considering the lower bound of ΔE00 (mean – standard deviation) of the distribution when determining the established thresholds. Measurements were performed in triplicate on three independent occasions (*n* = 9).

#### Water contact angle (WCA)

Contact angle readings were performed using a goniometer (Optical Contact Angle Measurements SCA20, DataPhysics Instruments GmbH, Germany). Static contact angles were measured using the sessile drop method, recorded and analyzed with the SCA20 software [37]. All droplets dispensed had the same volume (10 µL), standardized by the equipment. After the droplet reached the specimen surface, 5 s were established to allow stabilization and interaction of the liquid with the surface. Contact angles, formed between the surface of each specimen and the droplet (distilled water), were calculated using the Laplace-Young equation [37]. Measurements were performed after immersion times T0, T1, and T6, in two independent occasions with three specimens per group (*n* = 6).

#### Vickers hardness (HV)

Specimens of each group were evaluated using a Vickers diamond indenter, a validated tool for assessing hardness and viscoelastic responses of materials [38]. Measurements were performed with a Micromet 2100 (Buehler, USA). For conventional resin specimens, a load of 0.4 N was applied for 10 s. For 3D-printed resin specimens, previous tests were performed to adapt the protocol to a load of 0.9 N for 10 s to ensure adequate indentation. Diagonal lengths were measured immediately after indentation, within 10 s, to minimize viscoelastic recovery. Three indentations were performed per specimen and mean values were calculated. Measurements were performed in triplicate on three independent occasions (*n* = 9).

#### Surface roughness (Ra)

Surface roughness was measured at baseline and over time using a contact profilometer (Mitutoyo Corp. SJ 400, Japan). The specimens were analyzed with a resolution of 0.01 µm, interval of 0.8 mm, transverse length of 2.4 mm, speed of 0.5 mm/s, and diamond tip radius of 5 µm. Three readings were performed for each specimen and mean values were calculated. Measurements were performed in triplicate on three independent occasions (*n* = 9).

#### Scanning electron microscopy (SEM)

The surface topography of the specimens was evaluated by Scanning Electronic Microscopy (SEM) analysis. Specimens from each group (*n* = 1) were dehydrated, metallized with carbon and positioned in the microscope (JEOL JSM, 6610LV) to obtain the images. The final magnifications for each view were 100×, 250×, and 500×.

### Biological evaluation

#### *C. albicans* biofilm formation

Residual antimicrobial effect on the specimens was evaluated by analyzing biofilm adhesion and formation, through cell proliferation (colony-forming unit counts, CFU) and cell metabolic activity (AlamarBlue assay). Before testing, specimens of both resins were disinfected by exposure to UV light under dry conditions for 20 min each side [39].

*Candida albicans* (ATCC® 90028) was cultured on Sabouraud Dextrose Agar (SDA) at 37 °C for 48 h. Colonies were inoculated into Yeast Nitrogen Base (YNB) and incubated aerobically at 37 °C for 16 h. Subsequently, 0.5 mL of the culture was then transferred to 9.5 mL of fresh YNB and incubated for an additional 8 h. Cell density was adjusted spectrophotometrically at 540 nm, followed by centrifugation (10,000 rpm, 5 min), two phosphate-buffered saline (PBS) washes, and resuspension in RPMI-1640 medium to obtain standardized suspensions of 1 × 10⁶ CFU/mL. After disinfection, specimens were transferred to 24-well plates containing 750 µL of the *C. albicans* suspension and 750 µL of sterile RPMI-1640 (1.5 mL/well). Plates were incubated for 90 min at 37 °C under orbital shaking (75 rpm) to allow fungal adhesion. Non-adhered cells were removed by two PBS washes, fresh RPMI-1640 was added, and plates were incubated for 48 h at 37 °C to promote mature biofilm formation, with partial medium renewal after 24 h.

#### *C. albicans* biofilm proliferation and metabolism

After mature biofilm formation, the biofilm was detached by scraping with a sterile pipette tip for 1 min, serially diluted, and plated onto SDA supplemented with chloramphenicol (0.1 g/L). Plates were incubated at 37 °C for 48 h, after which colonies were counted, and CFU/mL was calculated using the formula (2):

2 $$\mathrm{CFU}/\mathrm{mL}=(\mathrm{Number\; of\; colonies})\,{\rm{x}}\,10{\rm{n}}/{\rm{q}}$$

In this formula, n is equivalent to the absolute value of the chosen dilution and *q* is equivalent to the quantity, in mL, of each dilution seeded on the plates. All procedures were performed in triplicate on three independent occasions per experimental group (*n* = 9).

Initially, for cell metabolism assay, *C. albicans* biofilm was formed on the specimens, as mentioned above. After 48 h, the mature biofilm was washed twice with 1000 µL of PBS. Then, 1500 µL of sterile RPMI-1640 was added to each well, followed by the addition of 150 µL of alamarBlue® solution (Invitrogen™, USA), following 10% (v/v) concentration. The plates were then placed in an orbital shaking incubator at 37 °C and 75 rpm for 4 h. After this period, 100 µL from each well were transferred in triplicate to a 96-well plate for fluorescence measurements using the Fluorstar Omega (Fluorstar Omega, BMG Labtech, Germany) with excitation/emission wavelengths of 560 nm (A560) and 590 nm (A590), respectively [40]. All procedures were performed in triplicate on three independent occasions (*n* = 9).

#### Residual cytotoxic effect

To assess cytotoxicity of extracts released by the specimens, cell metabolism analysis was performed by indirect exposure, following ISO 10993-5:2009 and ISO 10993-12:2021 [41, 42]. Mouse L-929 fibroblast line (NCTC clone 929 [L cell, L-929, derivative of strain L], CCL-1) was cultured in Dulbecco’s Modified Eagle Medium (DMEM) supplemented with 10% (v/v) fetal bovine serum (FBS), 1% (v/v) penicillin/streptomycin (10,000 µg/mL), amphotericin B (25 µg/mL), and 1 mM sodium pyruvate. Extracts were obtained by incubating three specimens previously disinfected from each group in 3 mL of DMEM at 37 °C for 24 h. The positive control consisted of 3 mL of medium stored under the same conditions.

For cytotoxicity testing, L-929 cells at 70% confluence were detached using 0.05% trypsin, centrifuged at 0.3 rcf for 5 min, and resuspended in 1 mL of medium for counting with an automated cell counter (Countess II FL; Life Technologies, USA). The suspension was adjusted to 1 × 10⁴ cells/well, and 100 µL was seeded into a 96-well plate and incubated at 37 °C for 24 h to allow adhesion. After this period, the culture medium was discarded, keeping the adherent cells, then 50 µL of fresh medium with 50 µL of the prepared extract were added to each well and incubated for 24 h. Afterward, 10 µL of AlamarBlue reagent was added to each well (10% v/v), and the plate was incubated for 4 h [43, 44]. Fluorescence was measured using a plate reader as previously described. All procedures were performed in triplicate on three independent occasions (*n* = 9). Results were expressed as percentage of cell metabolism relative to the positive control group, which was set as 100% metabolism, according to ISO 10993-5:2009 classification (see supplementary information, Table. S2).

### Statistical analysis

Due to the study design, all experiments were conducted using independent specimens at each time point. Accordingly, statistical analyses were performed using ANOVA tests for unpaired samples. Firstly, data were assessed for normality and homogeneity of variances using the Shapiro-Wilk and Levene’s tests, respectively. As these assumptions were met, a Three-way ANOVA (Resin × Solution × Time) was applied to evaluate main effects and interactions. When significant interactions were detected, analyses were further decomposed by Time, considering Resin × Solution as a single factor with eight independent groups and analyzed using Two-way ANOVA. Post hoc pairwise comparisons were performed using Tukey’s test. For additional interpretation of the results, marginal means were also presented by factor (Resin and Time). One-way ANOVA followed by Tukey’s post hoc test was applied to identify statistical differences within each factor. All statistical tests were performed at a significance level of 5% (*α* = 0.05).

Statistical analyses were carried out using SPSS for Windows® (IBM® SPSS® Statistics, version 29.0.0.0, USA) and GraphPad Prism® (version 10.5.0, GraphPad Software, USA). Graphs were generated using GraphPad Prism®, and figures were created with Inkscape (version 1.4.3, Inkscape Project) and BioRender.com.

## Results

### Physical evaluation

#### Color change

**Fig. 1:**
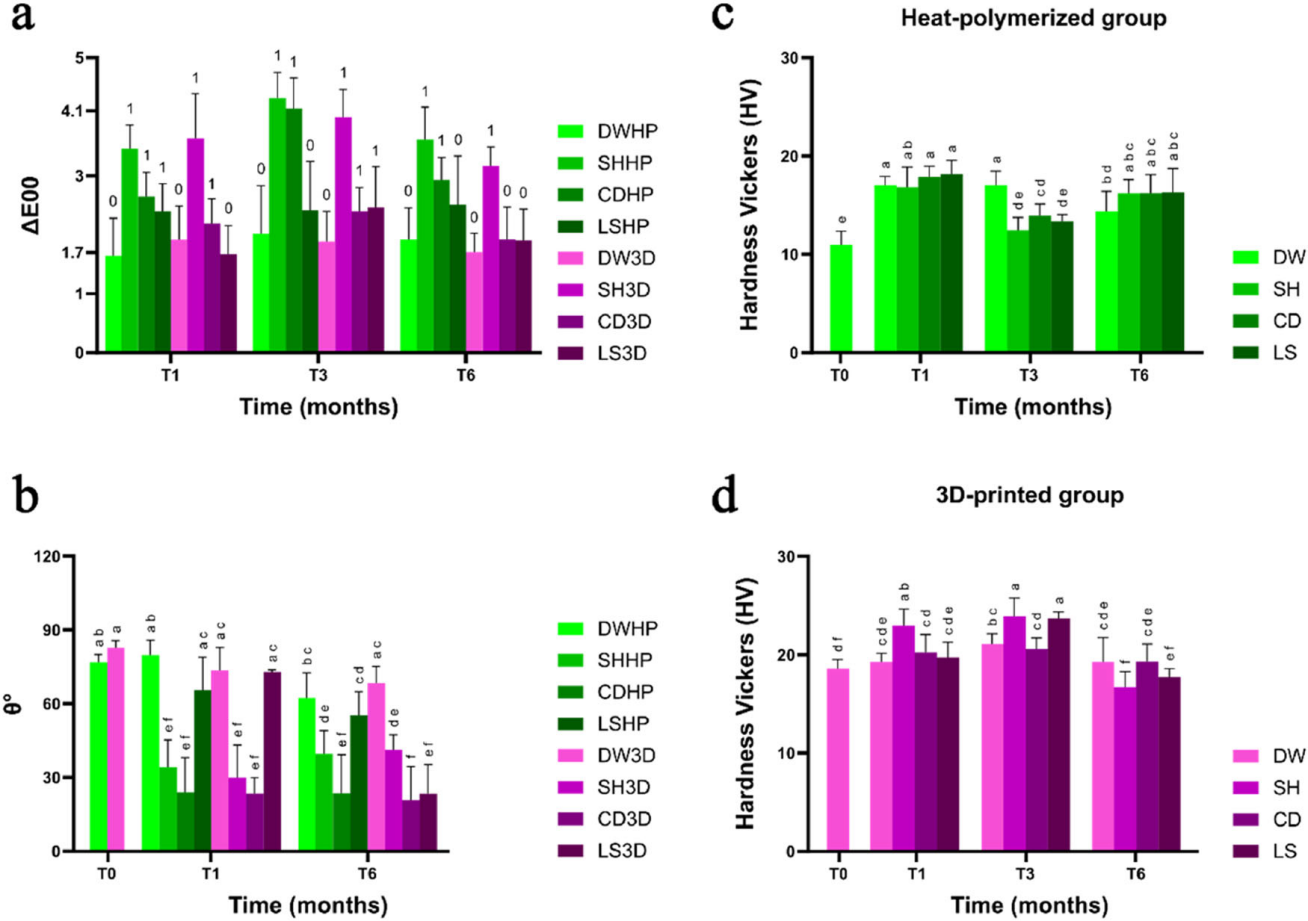
Physical evaluation of the investigated groups of heat-polymerized (HP) and 3D-printed resins (3D) immersed in the solutions namely distilled water (DW), 1% sodium hypochlorite (SH), 2% chlorhexidine digluconate (CD), and soap solution (LS, Lifebuoy®), at zero or baseline (T0), one (T1), three (T3), and six (T6) months. **a** Color change analysis (ΔE00) presented as mean ± standard deviation. *Note*. Numbers above the bars indicate the clinical classification based on the conservative lower bound (mean – standard deviation) of ΔE00, according to Ren et al. [35]: (0) Clinically imperceptible changes (ΔE00 ≤ 1.7); (1) Clinically acceptable changes (ΔE00 = 1.7–4.1); (2) Clinically unacceptable changes (ΔE00 ≥ 4.1). **b** Water contact angle (θ°) shown as mean ± standard deviation. **c** Vickers hardness (HV) (mean ± standard deviation) values of heat-polymerized resin group **d** Vickers hardness (HV) (mean ± standard deviation) values of 3D-printed group. *Note*. Different lowercase letters above bars denote statistically significant differences between groups (Tukey’s test, *p* < 0.05)

Three-way ANOVA showed statistically significant effects for resin type, disinfectant solutions, and immersion time, as well as their interactions (see supplementary information, Table S3). Figure 1a and Table. S4 (see supplementary information) showed that SH and CD in heat-polymerized specimens resulted in the highest ΔE00 values at T3 (4.3 ± 0.4 and 4.1 ± 0.5, respectively). However, when interpreted by ΔE00 mean – SD, neither group was classified as clinically unacceptable according to Ren et al. [36].

For 3D-printed specimens, SH produced ΔE00 values of approximately 3.0 from T1 to T6, which were classified as clinically acceptable color changes. Immersion in Lifebuoy soap resulted in variations in ΔE00 over time but presented values comparable to the control group at T6 and was classified as clinically imperceptible in both resins after 6 months.

#### Water contact angle

Three-way ANOVA revealed no difference in hydrophobicity between 3D-printed and conventional resins, but significant effects were found for solutions, immersion time, and interactions (see supplementary information, Table S5). Figure 1b showed that immersion in SH and CD for 1 and 6 months significantly reduced the contact angle of heat-polymerized specimens, reaching values of approximately 36.5° ± 9.5° and 23.0° ± 14.5°, respectively, compared with baseline specimens (76.8° ± 3.0°). Immersion in LS showed no significant differences compared with the control group (DWHP).

For 3D-printed resin, significant reductions in contact angle were also observed at T1 in SH and CD, with values of approximately 35.0° and 21.5°, respectively, compared with baseline specimens (82.7° ± 2.8°). After 6 months, all tested solution groups (SH3D, CD3D, and LS3D) showed similar contact angle values and were statistically different from the control group (DW3D).

#### Vickers hardness

Hardness was analyzed by Two-way ANOVA, with separate analyses performed for each resin group. Heat-polymerized resin showed lower mean values (14.6 ± 2.9 HV) than 3D-printed resin (19.9 ± 2.4 HV). For PMMA specimens, significant effects were observed only for immersion time and their interaction with disinfectant solutions (see supplementary information, Table S6). Figure 1c showed that all solutions produced a similar pattern, with increased hardness at T1 and recovery by T6 with values significantly similar to T1. Overall, all disinfectant solutions led to increased hardness in heat-polymerized specimens compared with baseline.

For 3D-printed resin, as shown in Fig. 1d, significant effects were found for disinfectant solutions and immersion time, as well as their interactions (see supplementary information, Table S7). In this group, hardness values remained stable across all disinfectant solutions. While SH3D showed greater variations over time, CD3D and LS3D were comparable to the control group (DW3D) at T1, T3, and T6, with no statistically significant differences.

#### Surface roughness

**Fig. 2:**
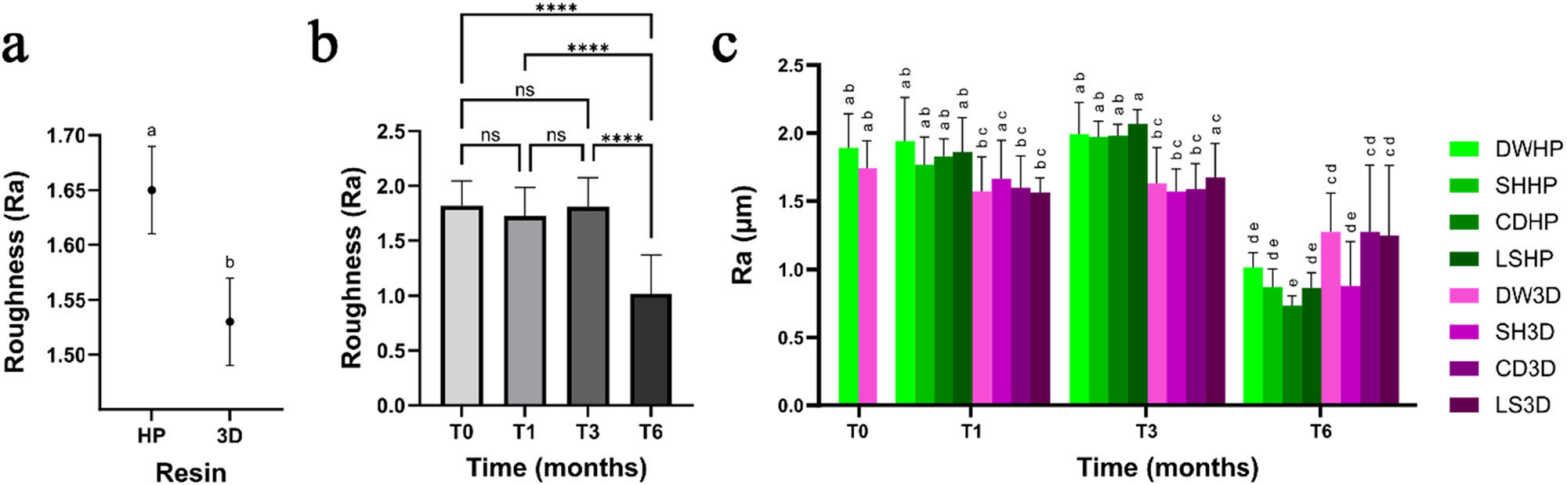
Surface roughness (Ra, µm) of the investigated groups of heat-polymerized (HP) and 3D-printed resins (3D) immersed in the solutions namely distilled water (DW), 1% sodium hypochlorite (SH), 2% chlorhexidine digluconate (CD), and soap solution (LS, Lifebuoy®), at zero or baseline (T0), one (T1), three (T3), and six (T6) months. **a** Ra according to the groups of resin (mean ± standard error of the mean). **b** Ra according to the immersion times (mean ± standard deviation). **c** Ra according to the interaction between solution and resin over time (mean ± standard deviation). *Note*. Different lowercase letters above bars denote statistically significant differences between groups (Tukey’s test, *p* < 0.05). Asterisks above bars indicate significant differences (**** *p* < 0.0001) and “ns” denotes non-significant difference

Three-way ANOVA indicated statistically significant effects only for the isolated factors resin type and immersion time, as well as for their interaction (see supplementary information, Table S8). Figure 2 presented surface roughness (Ra) values and methodology was illustrated in the supplementary information (Fig. S9). Heat-polymerized group showed a mean Ra of 1.6 ± 0.4 µm, which was significantly different from the 3D-printed resin with 1.5 ± 0.3 µm (Fig. 2a). Regarding roughness over time, mean values were statistically similar at T0, T1, and T3, but a significant reduction was observed at T6 (Fig. 2b). Considering the interaction between solution and resin over time, Fig. 2c showed that roughness remained stable from baseline (T0) to 3 months, followed by a significant reduction at T6 in both resins. This effect was more pronounced in heat-polymerized specimens for all solutions, which reached values below 1.0 µm.

#### Scanning electron microscopy analysis

**Fig. 3:**
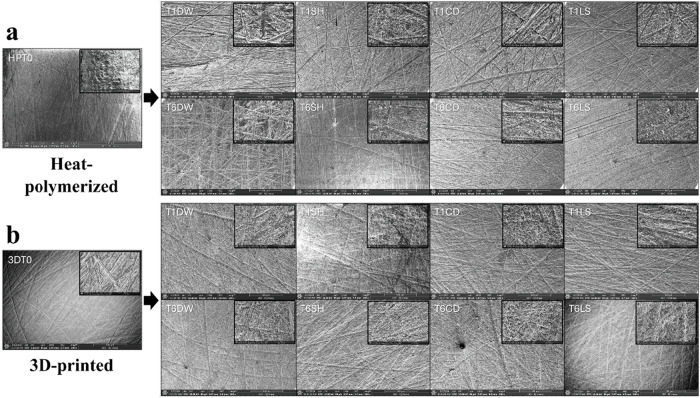
Scanning electron microscopy images of heat-polymerized (HP) and 3D-printed resins (3D) immersed in the solutions namely distilled water (DW), 1% sodium hypochlorite (SH), 2% chlorhexidine digluconate (CD), and soap solution (LS, Lifebuoy®), at zero or baseline (T0), one (T1), three (T3), and six (T6) months. **a** Topographical surface of HP specimens. **b** Topographical surface of 3D specimens. *Note*. Representative micrographs were shown at 100× (main images) and 500× (right insets) magnifications

SEM images displayed in Fig. 3 revealed irregular and heterogeneous finishing marks in heat-polymerized resin at baseline, whereas 3D-printed specimens showed a more striated pattern (Fig. 3a, b). When immersed in distilled water, heat-polymerized specimens exhibited a fibrillar organization that progressively degraded over time, while 3D-printed specimens showed a denser and bulk-like structure. From T1 to T6, heat-polymerized specimens demonstrated surface smoothing after exposure to disinfectant solutions. 3D-printed specimens exhibited structural changes characterized by erosion and porosity, particularly in the CD and LS groups. Additional SEM images with different magnifications were provided in the supplementary information (Figs. S10.1–5).

### Biological evaluation

**Fig. 4:**
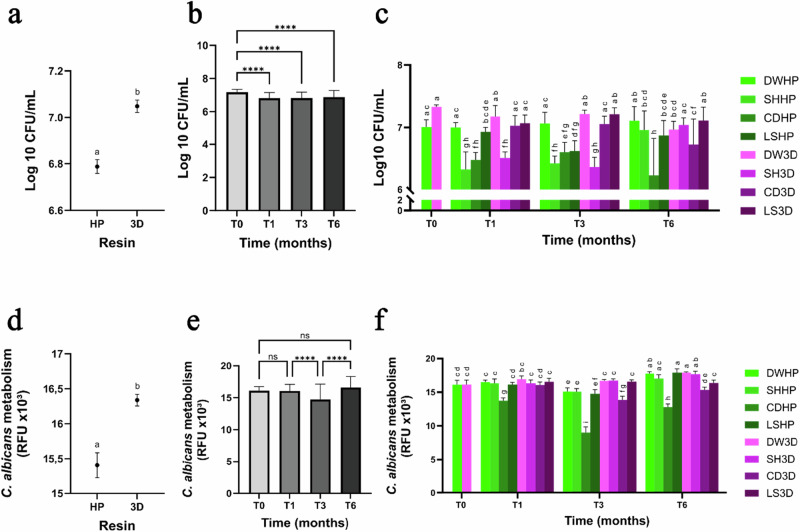
Residual antimicrobial effect evaluated by *C. albicans* proliferation (Log 10 CFU/mL) and *C. albicans* biofilm metabolism (RFU) tests of the investigated groups of heat-polymerized (HP) and 3D-printed resins (3D) immersed in the solutions namely distilled water (DW), 1% sodium hypochlorite (SH), 2% chlorhexidine digluconate (CD), and soap solution (LS, Lifebuoy®), at zero or baseline (T0), one (T1), three (T3), and six (T6) months. **a** Log10 CFU/mL values according to the groups of resin (mean ± standard error of the mean). **b** Log10 CFU/mL values according to the immersion times (mean ± standard deviation). **c** Log10 CFU/mL values according to the interaction between solution and resin over time (mean ± standard deviation). **d** RFU values according to the groups of resin (mean ± standard error of the mean). **e** RFU values according to the immersion times (mean ± standard deviation). **f** RFU values according to the interaction between solution and resin over time (mean ± standard deviation). *Note*. Different lowercase letters above bars denote statistically significant differences between groups (Tukey’s test, *p* < 0.05). Asterisks above bars indicate significant differences (**** *p* < 0.0001) and “ns” denotes non-significant difference

The CFU/mL and cell metabolism data were analyzed using Three-way ANOVA, which demonstrated significance of the three independent factors and their interations (see supplementary information, Tables S11 and S12). Figure 4 presented the results of Log10 CFU/mL and *C. albicans* biofilm metabolic activity in Relative Fluorescence Units (RFU). The methodology was illustrated in the supplementary information (Fig. S13).

#### *C. albicans* proliferation (CFU)

Results showed that *C. albicans* adhesion and biofilm formation was significant higher in 3D (7.0 ± 0.0 log) compared to HP specimens (6.7 ± 0.0 log) (Fig. 4a). When time was analyzed, overall CFU values significantly decreased at T1, T3, and T6 compared to baseline (T0) (Fig. 4b). Among disinfectants, CD consistently resulted the greatest reductions in CFU, as for CDHP and CD3D specimens, mean ± SD after 6 months of immersion were 6.2 ± 0.5 log and 6.7 ± 0.4 log, respectively, followed by SH and LS, which demonstrated intermediate activity with CFU values around 7.0 log (Fig. 4c).

#### *C. albicans* biofilm metabolism (AlamarBlue assay)

Similar to CFU results, higher metabolic activity was observed in 3D compared to HP specimens (Fig. 4d). Regarding immersion time, metabolic activity decreased significantly at T3 compared with baseline, although no significant difference was found between T0 and T1, or T0 and T6 (Fig. 4e). Analysis of the disinfectant solutions over time demonstrated the lowest fungal metabolic activity recorded in biofilms of CDHP group after 3 and 6 months (9011.2 ± 837.8 a.u. and 12,759.2 ± 485.1 a.u., respectively) (Fig. 4f). In 3D group, the solutions were statistically similar to DW3D over time, except for CD3D at T3 and T6 and LS3D at T6.

#### Cytotoxicity

Results were expressed as mean ± standard deviation of % cell metabolism relative to the positive control group and classified according to ISO 10993-5:2009 [40]. HP specimens immersed in CD showed moderate cytotoxicity, with a marked reduction in metabolism after 3 months (45.2% ± 6.3%). After 6 months, cell metabolism increased to 70.8% ± 1.9%, reaching non-cytotoxic levels. Similarly, 3D specimens immersed in CD exhibited slight cytotoxicity at T1 and T3, but resulted in non-cytotoxicity after 6 months (see supplementary information, Table S14).

**Fig. 5:**
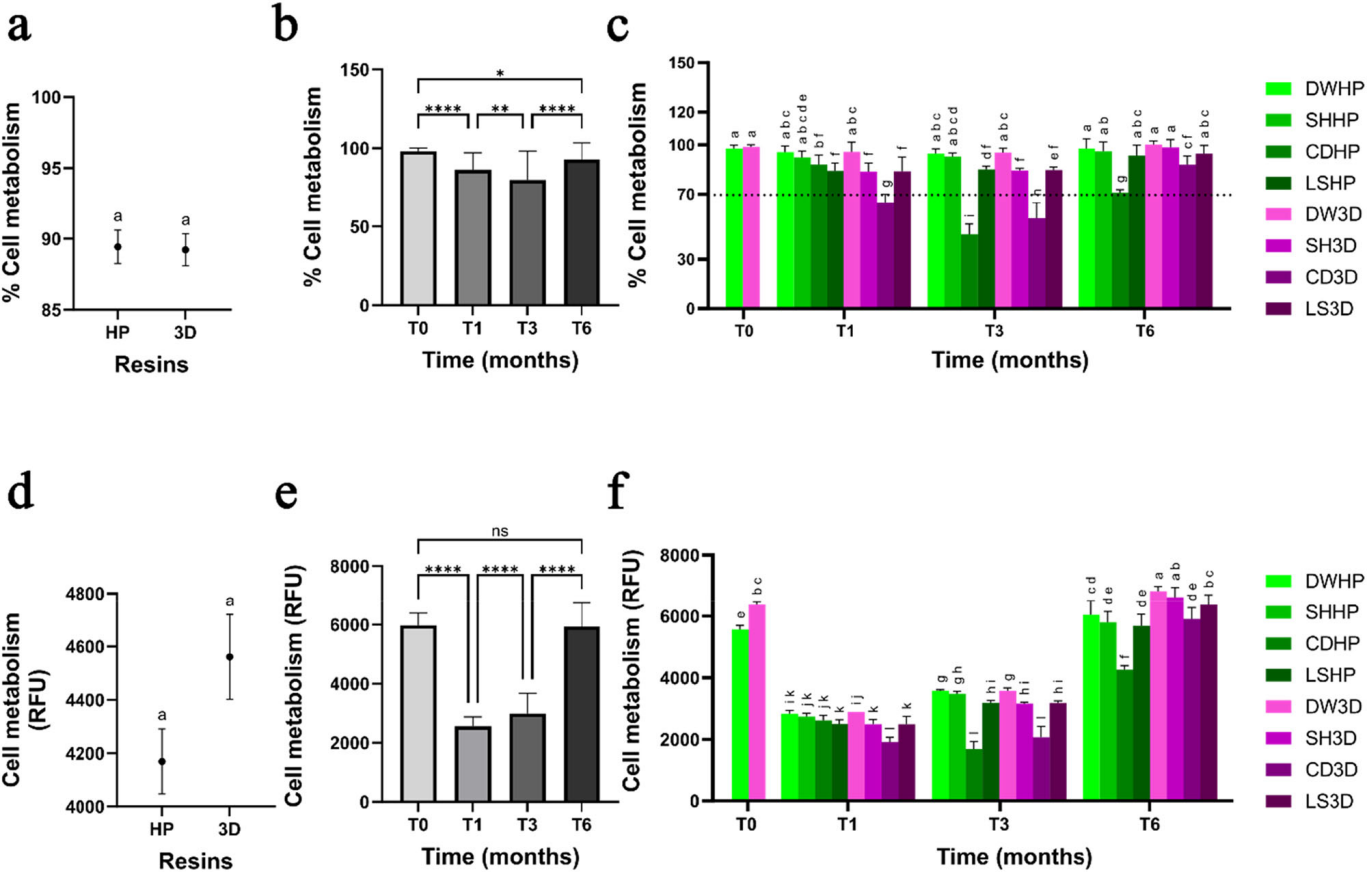
Residual cytotoxicity effect evaluated by percentage of L-929 cell metabolism (RFU) after indirect exposure test of the investigated groups of heat-polymerized (HP) and 3D-printed resins (3D) immersed in the solutions namely distilled water (DW), 1% sodium hypochlorite (SH), 2% chlorhexidine digluconate (CD), and soap solution (LS, Lifebuoy®), at zero or baseline (T0), one (T1), three (T3), and six (T6) months. **a** % Cell metabolism values according to the groups of resin (mean ± standard error of the mean). **b** % Cell metabolism values according to the immersion times (mean ± standard deviation). **c** % Cell metabolism values according to the interaction between solution and resin over time (mean ± standard deviation). *Note*. The dashed line marks the 70% ISO threshold for non-cytotoxicity. **d** RFU values according to the groups of resin (mean ± standard error of the mean). **e** RFU values according to the immersion times (mean ± standard deviation). **f** RFU values according to the interaction between solution and resin over time (mean ± standard deviation). *Note*. Different lowercase letters above bars denote statistically significant differences between groups (Tukey’s test, *p* < 0.05). Asterisks above bars indicate significant differences (* *p* ≤ 0.0332, ** *p* ≤ 0.0021, **** *p* < 0.0001) and “ns” denotes non-significant difference

The results were also analyzed using Three-way ANOVA, which revealed significance of the three independent factors and their interations (see supplementary information, Tables S15 and S16). Figure 5 showed the distribution of % and RFU values for cell metabolism obtained by AlamarBlue assay. The methodology was illustrated in the supplementary information (Fig. S17). The % cell metabolism and RFU values did not differ significantly between resin groups (Fig. 5a, d). Both analyses showed a similar temporal trend, with increased cytotoxicity from T1 to T3 that normalized by T6 compared to T0 (Fig. 5b, e).

When analyzing the interaction between solution and resin over time, CD immersion resulted in the highest reduction of cell metabolism (Fig. 5c, f). In the heat-polymerized group, CDHP showed a statistically significant reduction compared with DWHP at T3 and T6. Similarly, in the 3D-printed group, CD3D also exhibited significant decrease of cell metabolism compared with DW3D at T1, T3, and T6. In general, specimens from groups LSHP, LS3D, SHHP, and SH3D presented statistically similar RFU values across time points, although these values remained significantly lower than DWHP and DW3D. For example, LSHP showed mean values of 5,573.5 ± 122.1 a.u. at baseline and 5,679.8 ± 378.3 a.u. at T6, while LS3D showed 6,380.2 ± 89.7 a.u. at baseline and 6,363.1 ± 336.2 a.u. at T6 (Fig. 5f).

## Discussion

This study evaluated the effects of a 6-month disinfection protocol on heat-polymerized PMMA and 3D-printed denture base resins. The results showed material-specific differences regarding surface characteristics, residual antimicrobial, and cytotoxicity effect over time. Consequently, the null hypothesis was rejected, as long-term disinfection significantly altered both physical and biological properties of the tested resins.

### Physical evaluation

The results showed that sodium hypochlorite (SH) and chlorhexidine digluconate (CD) induced the greatest color changes over time, although all values remained clinically acceptable for both resins. These findings contrast with studies [24, 34] that reported significant differences after immersion in comparable solutions, even though their protocols were limited to 21 days [24] or simulated immersion of 6 and 12 months [34]. Consistent with the present investigation, Elhagali et al. [45] observed no clinically significant color change in 3D‑printed specimens immersed in 1% SH, despite the shorter immersion protocol (30 min). Such discrepancies may be attributed to variations in immersion protocols, color assessment standards, and the oxidative or cationic properties of the solutions, which can generate chromophores or degrade pigments in the polymer matrix [45, 46].

Moreover, the differences in color stability between additively manufactured resins and conventional PMMA may stem from their distinct chemical compositions and resulting polymer network structures, which influence cumulative solution exposure and pigment diffusion [47]. Notably, an increase in ΔE00 was observed at the 3-month time point for both groups, though slightly higher in PMMA. This behavior can be explained by an initial phase of solution sorption, followed by matrix saturation and stabilization, usually occurring within 7–60 days depending on monomer chemistry [48, 49]. Furthermore, given the long-term nature of the study, minor fluctuations may be due to variability in specimen positioning during repeated measurements.

Overall, 3D-printed specimens presented higher hardness than PMMA and relatively stable over 6 months, while PMMA showed variations and an increase after 6 months compared to baseline. This observation aligns with a previous study that reported an initial decrease in hardness of PMMA specimens after chemical disinfection, followed by recovery after 2 months of immersion in water [50]. In contrast, other investigations described lower hardness values for similar 3D printing denture base resin compared with heat-polymerized PMMA after thermocycling, as well as a reduction in hardness after 180 days of immersion, including in distilled water [51, 52]. The difference between the studies can be explained by the composition of the materials. For example, urethane acrylate-based 3D-printed denture base resin can exhibit higher hardness values [53].

The decrease in hardness and other properties may result from the separation of polymer chains by solvents within the network, a process known as plasticizing. The rate of this softening matches solvent uptake until the network is fully saturated [48]. Although water uptake was not directly tested, the literature reports that 3D-printed denture base resins exhibit lower water sorption and solubility compared with heat-cured acrylics [54], while still complying with ISO requirements [55]. Dimensional changes of a polymer network in a solvent are complex, difficult to predict, and highly material-dependent [48]. Additionally, the effect of disinfectants must be considered: sodium hypochlorite, with its alkaline pH and chlorine content, can exert a plasticizing effect that compromises stability; likewise, chlorhexidine, with its cationic properties, and Lifebuoy, even at high dilution with its acidic pH, may reduce polymer interchain interactions over time.

Regarding surface roughness, the finishing procedure prior to immersion explains the equal baseline results. However, processing methods can affect polymer structure, as in PMMA, bubbles may be trapped during flasking [56, 57], whereas 3D printing yields smoother finishes through a standardized workflow [58, 59]. Printing parameters also influence topography. The 90° building orientation was adopted due to its lower roughness compared with 0° and 45° [29]. After 6 months, Ra values decreased for both materials, contrasting with reports of no changes over shorter periods (up to 28 days) in PMMA [24] or increased Ra in a similar 3D printing resin [60]. These differences are possibly due to chemical smoothing effect of disinfectants during long-term immersion, dissolving surface irregularities [61, 62], which may not be evident under short-term or simulated conditions. In contrast, Amaya Arbeláez et al. [63] reported increased roughness in PMMA after 1, 3, and 6 months of immersion, likely due to differences in the disinfectant solutions used in the present study.

Wettability resulted in similar WCA at baseline between heat-polymerized and 3D-printed resins. In the study of Poker et al. [64], the conventional resin had higher contact angle than the 3D-printed resin (84° vs. 61°). This property is largely determined by monomer chemistry and polymerization linkages. Commercial composites have been formulated with monomers such as Bis-EMA, Bis-MA, BisPMA, EGDMA (ethylene glycol dimethacrylate), TE-EGDMA (tetraethylene glycol dimethacrylate), and other dimethacrylates, whose resultant polymers are not considered highly hydrophilic [48]. Although the literature provides valuable insights into monomer hydrophilicity, the lack of detailed information about the resin compositions used in this study limited direct comparisons.

According to the Wenzel model [65], surface roughness can influence wettability depending on the intrinsic properties of the material. In the study by Poker et al. [64], specimens were polished to ensure standardization, which may explain why, despite PMMA presenting higher WCA, the values still fell within the hydrophilic range. Similarly, in the present study, both 3D-printed and conventional PMMA specimens were finished and exhibited hydrophilic behavior at baseline, consistent with the Wenzel model. Disinfection further amplified this effect, with SH and CD significantly increasing surface hydrophilicity, in line with findings that 3D-printed resin showed lower WCA after immersion in 1% SH [66]. These changes likely reflect the solutions’ effects, such as oxidation and increased polarity, which promote water spreading over the specimens [67].

Residual monomer may also affect the physical and mechanical properties of denture base materials. Although this study did not evaluate this parameter, previous investigations have shown considerably lower degrees of conversion in acrylic-based photopolymers compared to conventional resins [68]. Manufacturers also claim that 3D-printed materials release up to six times less monomer than PMMA [69]. Properly processed heat-polymerized PMMA, however, is expected to release minimal amounts. In one study [55], heat-polymerized PMMA complied with ISO 1567 requirements, particularly when dentures were immersed in water at 37 °C for 1 or 2 days before insertion [70]. As this protocol was followed in the present study, caution was warranted in attributing the observed modifications solely to residual monomer release.

The findings indicate that the physical and mechanical variations observed were more likely associated with fundamental differences in chemical composition. PMMA is typically produced by polymerization of methyl methacrylate (MMA) monomers in the presence of benzoyl peroxide [71]. In contrast, Penzenstadler et al. [72] identified 63 compounds in 3D-printing resins, including MMA derivatives, photoinitiators, UV stabilizers, and additives. The presence of high MMA concentrations suggests a potential risk of monomer elution, depending on the degree of conversion and duration of tissue contact. Ethyl-4-dimethylaminobenzoate, a photoinitiator detected in these resins, enhances curing but is known to cause irritation. Moreover, the identification of undisclosed compounds raises safety concerns and limits direct comparison with other photopolymers and PMMA.

### Biological evaluation

Colonization of denture surfaces by *C. albicans* is considered a major contributing factor for DS development [10]. Biofilm formation on material surfaces is influenced by several factors, including wettability, roughness, chemical composition, and surface charge [12, 73, 74]. Higher roughness (Ra > 0.2 μm) typically facilitates microbial adhesion [75, 76], while hydrophilic surfaces (*θ* < 90°) [77] and negatively charged interfaces are often linked to reduced biofilm development. In the context of 3D printing, variables such as building orientation and post-curing can significantly alter surface characteristics and microbiological behavior [78]. In the present study, the 90° printing orientation was selected to minimize both roughness and *C. albicans* adhesion [29], although this was not observed in the present results.

The relationship between roughness and microbial adhesion is classically controversial. In this study, there was no significant association between surface roughness and *C. albicans* biofilm formation. This agrees with Izumida et al. [79], although other studies reported more fungal adhesion on rougher surfaces [40, 80, 81]. Despite the overall higher Ra in heat‑polymerized specimens, *C. albicans* growth and metabolism were greater on 3D‑printed resins. Poker et al. [64] reported that surface roughness did not influence microbial counts in 3D‑printed resins, since all tested materials were within the clinically acceptable range (Ra < 0.2 µm), and even under these conditions, *S. mutans* and *S. aureus* adhered more to conventional resins than to 3D‑printed ones, with surface free energy and wettability identified as the main determinants of microbial adhesion.

Thus, substrates with a high polar component or hydrophobicity favor microbial colonization through non-covalent interactions with cell wall proteins (e.g., Csh1) [73, 82], as well as by the interfacial free energy of liquids and interactive surfaces [83]. In this study, 3D-printed specimens showed greater fungal colonization than conventional group, even under the residual effects of disinfectants that increased surface hydrophilicity, indicating that polymer characteristics were more decisive in shaping microbial behavior. In addition, Poker et al. [64] did not find significant differences in *C. albicans* adhesion between conventional (hydrophobic) and 3D-printed (hydrophilic) resin surfaces, suggesting that wettability alone may not be a critical factor for adhesion, and consistent with previous studies attributing higher *C. albicans* colonization to the intrinsic properties of 3D printing resins [6, 84].

Evidence from previous studies indicates that microbial adhesion and biofilm formation are influenced by manufacturing methods and materials, with higher adhesion of *C. albicans* on 3D‑printed surfaces [6, 84, 85]. Consistent with the results, Koujan et al. [85] observed significantly less *C. albicans* adherence on heat-cured PMMA compared with 3D-printed PMMA. This was further confirmed by Meirowitz et al. [6], who showed that 3D printing significantly increased microbial cell adhesion compared with heat curing, while CAD-CAM milling reduced adhesion. Taken together, the literature indicates that different manufacturing techniques create distinct surface conditions that either facilitate or hinder fungal colonization. Supporting this, virulence-related processes, including *C. albicans* biofilm formation, hyphal transition, and extracellular matrix production have been reported more frequently in 3D-printed specimens than in milled and heat-polymerized groups [84].

Residual antimicrobial activity from the solutions influenced the growth and metabolism of *C. albicans* under all conditions. The effectiveness of chlorhexidine in both groups may be attributed to its cationic nature, which disrupts yeast cell membranes and exerts a fungicidal effect. In addition, its substantivity allows the compound to be absorbed into the material, maintaining antimicrobial activity through gradual release and daily renewal [28, 60, 86]. Although less pronounced than that of CD, LS also prevented *C. albicans* colonization while preserving physical properties. This observation is consistent with Tasso et al. [25], who reported that daily overnight immersion significantly reduced the presence of two *Candida* species in denture wearers after 7 days. SH also showed antifungal activity, but higher concentrations are often associated with surface damage and discoloration [28, 61, 87], reinforcing the need to balance antimicrobial efficacy and material integrity.

Residual cytotoxicity testing showed that most disinfectant solutions were non-cytotoxic to mouse fibroblasts according to ISO 10993-5:2009 classification. However, immersion in CD for 1 and 3 months resulted in slight to moderate cytotoxicity, likely due to its strong ability to impregnate in the material, related to substantivity, and its higher concentration compared with SH [27, 28, 60]. This time-dependent pattern, with increased toxicity at intermediate intervals and partial recovery after 6 months, aligns with the dynamics of solution uptake and saturation within the polymer structure [48]. CD exposure has also been shown to produce toxic effects in human oral and dermal cells even at sub-clinical concentrations [88, 89]. Conversely, LS and 1% SH remained non-cytotoxic over time, consistent with findings that 0.5% SH did not affect keratinocyte cell metabolism [32].

In summary, 3D-printed resins exhibited superior physical properties but greater *C. albicans* colonization than heat-polymerized materials, indicating a contrast between mechanical performance and biological behavior. The long-term immersion protocol was essential to reveal time-dependent changes in roughness and antimicrobial responses. Among the disinfectants, CD most effectively reduced biofilm formation, but presented residual cytotoxicity; SH was effective yet compromised color stability, while LS showed moderate antifungal action with favorable biocompatibility. Further clinical disinfection protocols may benefit from adjustments such as shorter exposure times, lower concentrations, or standardized rinsing to balance efficacy and material safety.

This study employed a controlled in vitro design to evaluate disinfection protocols on denture base resins after long‑term exposure. Nonetheless, certain limitations should be acknowledged in relation to clinical extrapolation. The use of a single‑species biofilm does not replicate the complexity of oral microbiota, mechanical brushing was not simulated, only a limited range of disinfection solutions was tested, and storage in distilled water may not fully reproduce intraoral conditions. Future research should incorporate polymicrobial biofilms, a broader spectrum of chemical agents, saliva‑analog media, combined mechanical and chemical cleaning, and in situ models to provide more clinically relevant recommendations for these materials.

## Conclusion

Long-term disinfection protocol altered both physical and biological properties of heat-polymerized PMMA and 3D-printed resins. While 3D-printed resins demonstrated superior physical performance, PMMA exhibited lower *Candida* colonization, indicating the contrast between mechanical and biological behavior.Regarding the tested solutions, chlorhexidine digluconate was identified as the most effective antibiofilm agent, though with residual cytotoxicity, and Lifebuoy® as a conservative and biocompatible alternative.

## Supplementary information

Supplementary information
