# Supplementary material for: Long-term disinfection of 3D-printed denture resin: physical and biological in vitro assessments
Source: J Mater Sci Mater Med. 2026 Apr 24;37(1):72. doi: 10.1007/s10856-026-07049-8 (PMC13246861; doi:10.1007/s10856-026-07049-8)

**Long-Term Disinfection of 3D-Printed Denture Resin: Physical and Biological In Vitro Assessments**

**Authors:** Amanda C. Ferro^a,b^, Caroline C. de Oliveira^c^, Bárbara L. Morais^a^, Jonatas S. de Oliveira^a^, Rodolfo D. Piazza^d^, Rodrigo F. C. Marques^d^, Carlos Mota^b^, Matthew B. Baker^e^, Janaina H. Jorge^a*^

**Affiliation:** ^a^Department of Dental Materials and Prosthodontics, School of Dentistry, São Paulo State University (Unesp), Araraquara, Brazil.

^b^Department of Complex Tissue Regeneration, Institute for Technology-Inspired Regenerative Medicine (MERLN), Maastricht University, Maastricht, The Netherlands.

^c^Department of Morphology, Genetics, Orthodontics and Pediatric Dentistry, School of Dentistry, São Paulo State University (Unesp), Araraquara, Brazil.

^d^Department of Analytical, Physico-chemistry and Inorganic Chemistry, Institute of Chemistry, São Paulo State University (Unesp), Araraquara, Brazil.

^e^Department of Instructive Biomaterials Engineering, Institute for Technology-Inspired Regenerative Medicine (MERLN), Maastricht University, Maastricht, The Netherlands.

***Corresponding Author**

Janaina Habib Jorge

São Paulo State University (UNESP), School of Dentistry

Department of Dental Materials and Prosthodontics

Humaita street, 1680 - Araraquara, São Paulo, Brazil

Phone: +55 16 3301-6550

E-mail: habib.jorge@unesp.br

**Acknowledgments**

The authors thank São Paulo Research Foundation (Fapesp) for supporting this study through grants 2021/09624-3, 2021/09555-1, 2021/11270-5, 2022/06884-7 and 2024/01648-9.

**Conflict of interest**

The authors declare no conflict of interest.

**Supplementary information**

Fig. S1 3D printing and conventional (flasking technique) workflows for obtaining the specimens. (a) Image processing in the slicing software FlashDLPrint V2.3.0. (b) Flashforge Hunter DLP printer used for sample fabrication. (c) Printing platform showing specimens oriented at 90°. (d) CiclOne unit used for cleaning with isopropanol under agitation followed by UV post-curing. (e) Heat-polymerized acrylic resin (Vipi Wave) manually mixed in a glass jar. (f) Metallic mold embedded in dental stone within the flask for specimen packing. (g) Hydraulic press used for flask closure, followed by polymerization in a microwave unit. (h) Finished specimens after deflasking and removal of excess material.


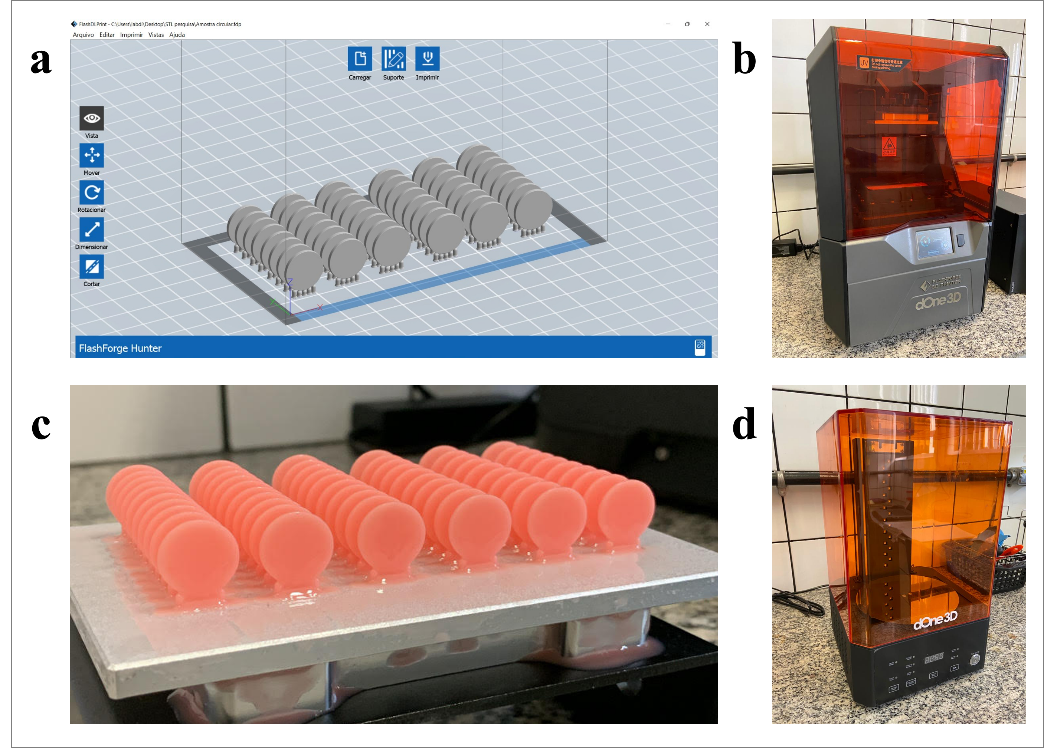


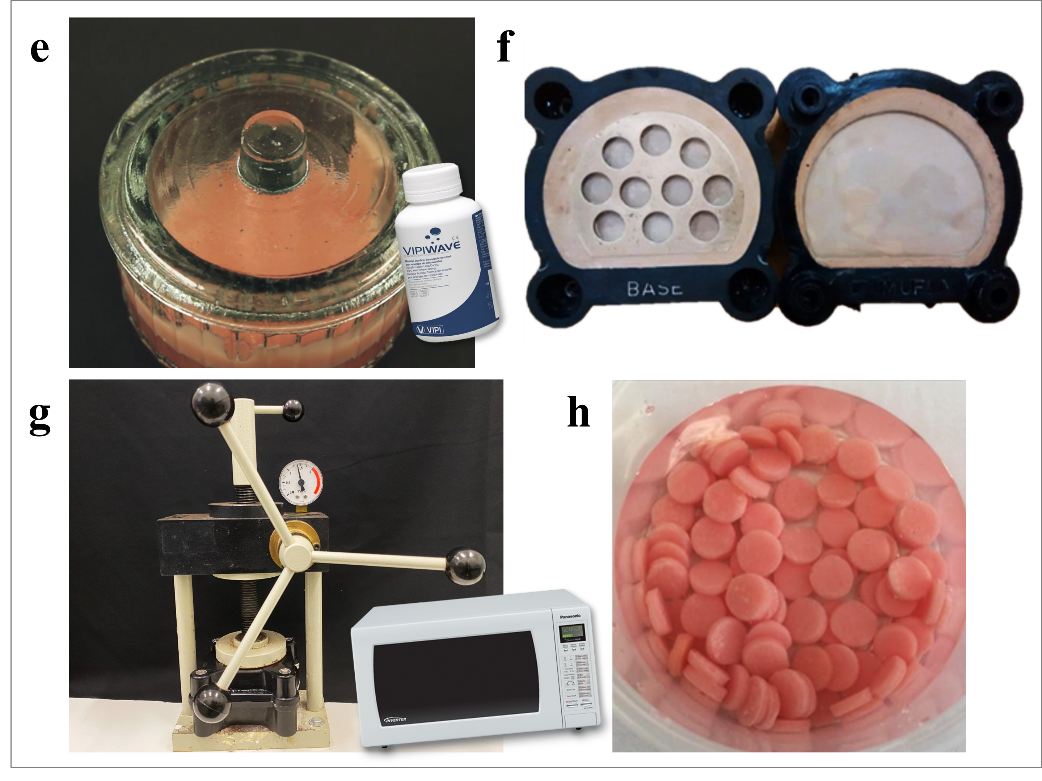


Table. S2 Classification of cytotoxicity according to ISO 10993-5:2009.

| **Cytotoxicity classification** | **Metabolism compared to control** | **Representation** |
| --- | --- | --- |
| Non-cytotoxic | > 70% | 0 |
| Slightly cytotoxic | 50-70% | 1 |
| Moderately cytotoxic | 30-50% | 2 |
| Severely cytotoxic | < 30% | 3 |

Table. S3 Three-way ANOVA for color change values calculated using CIEDE2000 (ΔE00). *Note*. Bold indicates statistically significant values (p < 0.05).

|  | **Sum of Squares** | **df** | **Mean Square** | **F** | **p** |
| --- | --- | --- | --- | --- | --- |
| Resin type | 10.08 | 1 | 10.078 | 32.85 | **< .001** |
| Solutions | 103.65 | 3 | 34.551 | 112.62 | **< .001** |
| Immersion time | 12.2 | 2 | 6.098 | 19.88 | **< .001** |
| Resin ✻ Solutions | 8.46 | 3 | 2.82 | 9.19 | **< .001** |
| Resin ✻ Time | 1.62 | 2 | 0.812 | 2.65 | 0.074 |
| Solutions ✻ Time | 4.19 | 6 | 0.699 | 2.28 | **0.038** |
| Resin ✻ Solutions ✻ Time | 5.27 | 6 | 0.879 | 2.86 | **0.011** |
| Residual | 58.91 | 192 | 0.307 |  |  |

Table. S4 Mean ± standard deviation values of ΔE00 for resin types, disinfectant solutions and immersion times. *Note*. Superscript numbers indicate the clinical classification based on the conservative lower bound (mean – standard deviation) of ΔE00, according to Ren et al. [35]: (0) Clinically imperceptible changes (ΔE00 ≤ 1.7); (1) Clinically acceptable changes (ΔE00 = 1.7–4.1); (2) Clinically unacceptable changes (ΔE00 ≥ 4.1).

| **Time** | | | | |
| --- | --- | --- | --- | --- |
| **Resin** | **Solution** | **T1** | **T3** | **T6** |
| **Heat-polymerized (HP)** | **DW** | 1.65 ± 0.63^0^ | 2.02 ± 0.81^0^ | 1.92 ± 0.54^0^ |
|  | **SH** | 3.46 ± 0.40^1^ | 4.32 ± 0.43^1^ | 3.62 ± 0.54^1^ |
|  | **CD** | 2.64 ± 0.41^1^ | 4.14 ± 0.51^1^ | 2.93 ± 0.37^1^ |
|  | **LS** | 2.40 ± 0.46^1^ | 2.41 ± 0.82^0^ | 2.51 ± 0.82^0^ |
| **3D-printed (3D)** | **DW** | 1.91 ± 0.57^0^ | 1.89 ± 0.50^0^ | 1.70 ± 0.32^0^ |
|  | **SH** | 3.63 ± 0.75^1^ | 3.99 ± 0.47^1^ | 3.16 ± 0.32^1^ |
|  | **CD** | 2.18 ± 0.42^1^ | 2.40 ± 0.39^1^ | 1.92 ± 0.54^0^ |
|  | **LS** | 1.67 ± 0.48^0^ | 2.47 ± 0.68^1^ | 1.90 ± 0.52^0^ |

Table. S5 Three-way ANOVA for water contact angle values (ϴ = º). *Note*. Bold indicates statistically significant values (p < 0.05).

|  | **Sum of Squares** | | **df** | | **Mean Square** | | **F** | | **p** | |  |
| --- | --- | --- | --- | --- | --- | --- | --- | --- | --- | --- | --- |
| Resin type | | 13.1 | | 1 | | 13.1 | | 0.169 | | 0.682 | |
| Solutions | | 21148.6 | | 3 | | 7049.5 | | 90.774 | | **< .001** | |
| Immersion time | | 38056.2 | | 2 | | 19028.1 | | 245.018 | | **< .001** | |
| Resin ✻ Solutions | | 371.9 | | 3 | | 124 | | 1.596 | | 0.194 | |
| Resin ✻ Time | | 975.5 | | 2 | | 487.7 | | 6.28 | | **0.003** | |
| Solutions ✻ Time | | 15347.7 | | 6 | | 2558 | | 32.938 | | **< .001** | |
| Resin ✻ Solutions ✻ Time | | 2581.1 | | 6 | | 430.2 | | 5.539 | | **< .001** | |
| Residual | | 9319.2 | | 120 | | 77.7 | |  | |  | |

Table. S6 Two-way ANOVA for hardness in the conventional heat-polymerized resin (HP). *Note*. Bold indicates statistically significant values (p < 0.05)

|  | **Sum of Squares** | **df** | **Mean Square** | **F** | **p** |
| --- | --- | --- | --- | --- | --- |
| Denture cleansers | 11.8 | 3 | 3.92 | 1.71 | 0.168 |
| Time | 827.3 | 3 | 275.76 | 120.19 | **< .001** |
| Cleansers ✻ Time | 130.6 | 9 | 14.51 | 6.33 | **< .001** |
| Residual | 293.7 | 128 | 2.29 |  |  |

Table. S7 Two-way ANOVA for hardness in the 3D-printed resin group (3D). *Note*. Bold indicates statistically significant values (p < 0.05)

|  | **Sum of Squares** | **df** | **Mean Square** | **F** | **p** |
| --- | --- | --- | --- | --- | --- |
| Denture cleansers | 20.3 | 3 | 6.77 | 3.42 | **0.019** |
| Time | 386.9 | 3 | 128.96 | 65.18 | **< .001** |
| Cleansers ✻ Time | 176.4 | 9 | 19.59 | 9.9 | **< .001** |
| Residual | 253.2 | 128 | 1.98 |  |  |

Table. S8 Three-way ANOVA for surface roughness values (Ra µm). *Note*. Bold indicates statistically significant values (p < 0.05).

|  | **Sum of Squares** | **df** | **Mean Square** | **F** | **p** |
| --- | --- | --- | --- | --- | --- |
| Resin type | 1.068 | 1 | 1.0682 | 18.289 | **< .001** |
| Denture cleansers | 0.326 | 3 | 0.1088 | 1.863 | 0.136 |
| Immersion time | 31.938 | 3 | 10.646 | 182.268 | **< .001** |
| Resin ✻ Cleansers | 0.124 | 3 | 0.0413 | 0.708 | 0.548 |
| Resin ✻ Time | 4.738 | 3 | 1.5794 | 27.04 | **< .001** |
| Cleansers ✻ Time | 0.496 | 9 | 0.0551 | 0.944 | 0.488 |
| Resin ✻ Cleansers ✻ Time | 0.734 | 9 | 0.0815 | 1.395 | 0.19 |
| Residual | 14.953 | 256 | 0.0584 |  |  |

Fig. S9 Surface roughness (Ra µm) of the investigated groups of heat-polymerized (HP) and 3D-printed resins (3D) immersed in the solutions namely distilled water (DW), 1% sodium hypochlorite (SH), 2% chlorhexidine digluconate (CD) and soap solution (LS, Lifebuoy®), at zero or baseline (T0), one (T1), three (T3) and six (T6) months. (a) Methodology used for measurement. (b) Ra according to the groups of resin (mean ± standard error of the mean). (c) Ra according to immersion times (mean ± standard deviation). (d) Ra according to interaction between solution and resin over time (mean ± standard deviation). *Note.* Different lowercase letters above bars denote statistically significant differences between groups (Tukey’s test, p < 0.05). Asterisks above bars indicate significant differences (**** p < 0.0001) and “ns” denotes non-significant difference.


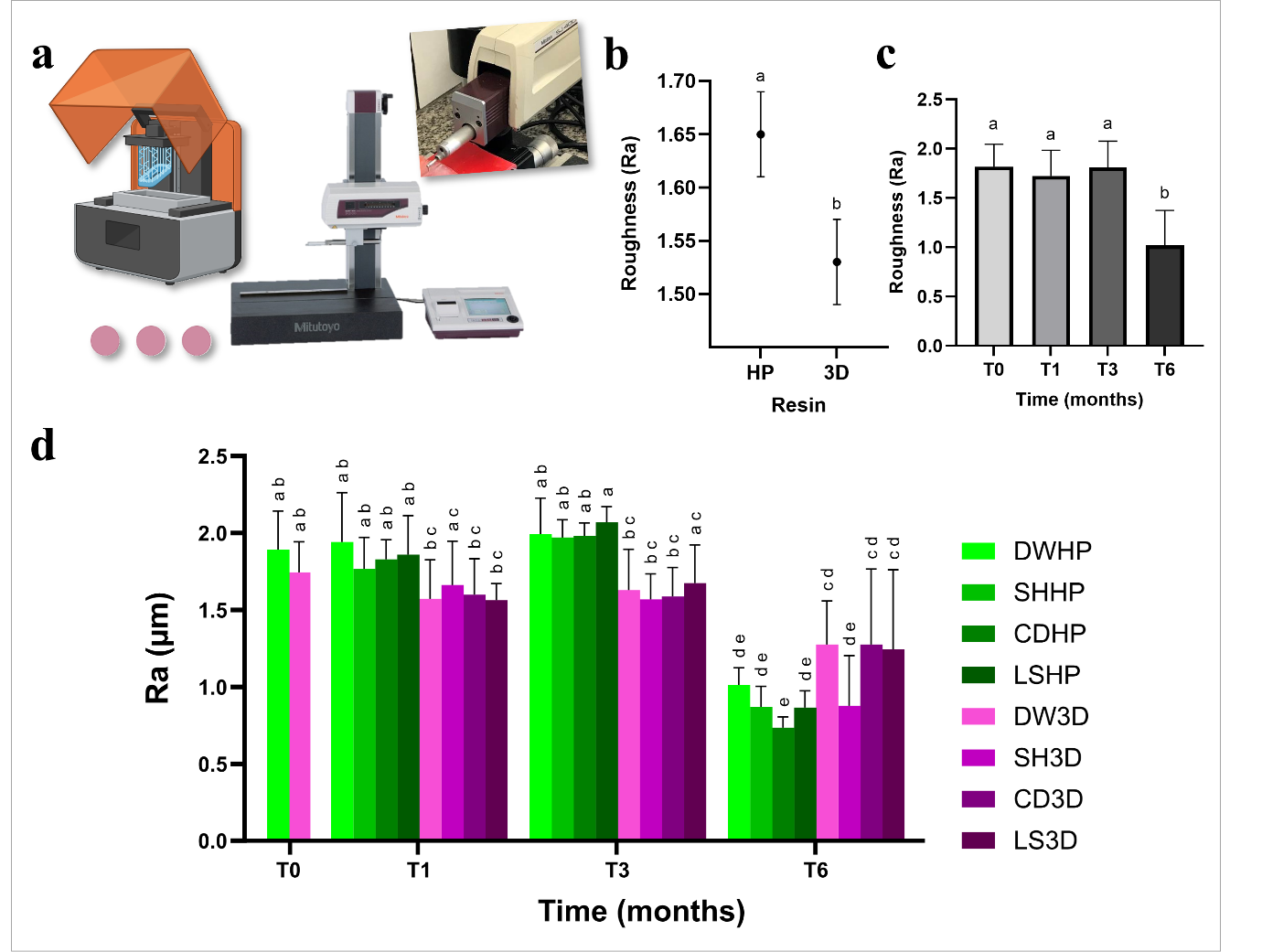


Fig. S10 Scanning electron microscopy (SEM) images of samples across different groups and magnifications:

Fig. S10.1 SEM micrographs of sample surfaces at baseline (T0) for both resin groups, conventional heat-polymerized (HP) and 3D-printed (3D), acquired at 100x, 250x and 500x, respectively. *Note*. T0 refers to specimens stored in distilled water for 48 h and used as the baseline.


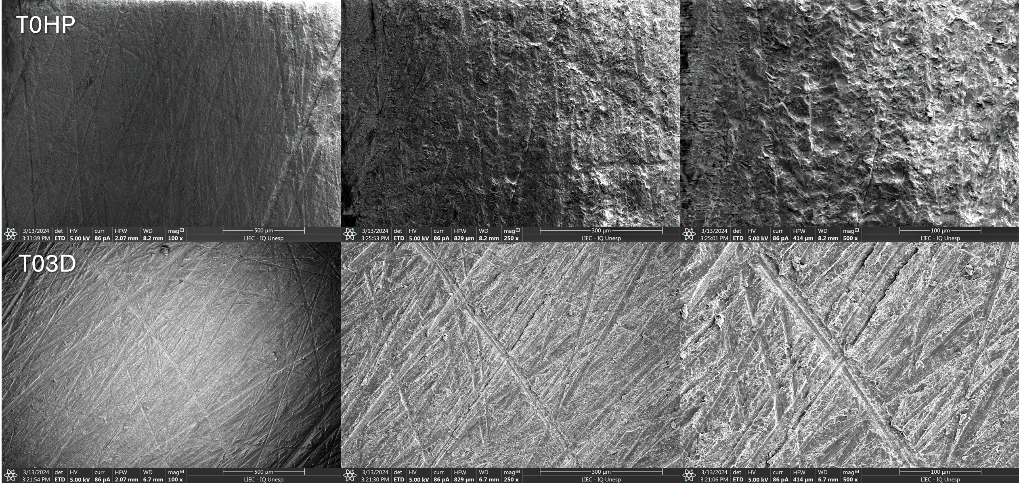


Fig. S10.2 SEM micrographs of sample surfaces immersed in distilled water (DW) at T1 and T6 for both resin groups, conventional heat-polymerized (HP) and 3D-printed (3D), acquired at 100x, 250x and 500x, respectively. *Note*. T1 and T6 refers to specimens immersed in the solutions for 1 and 6 months.


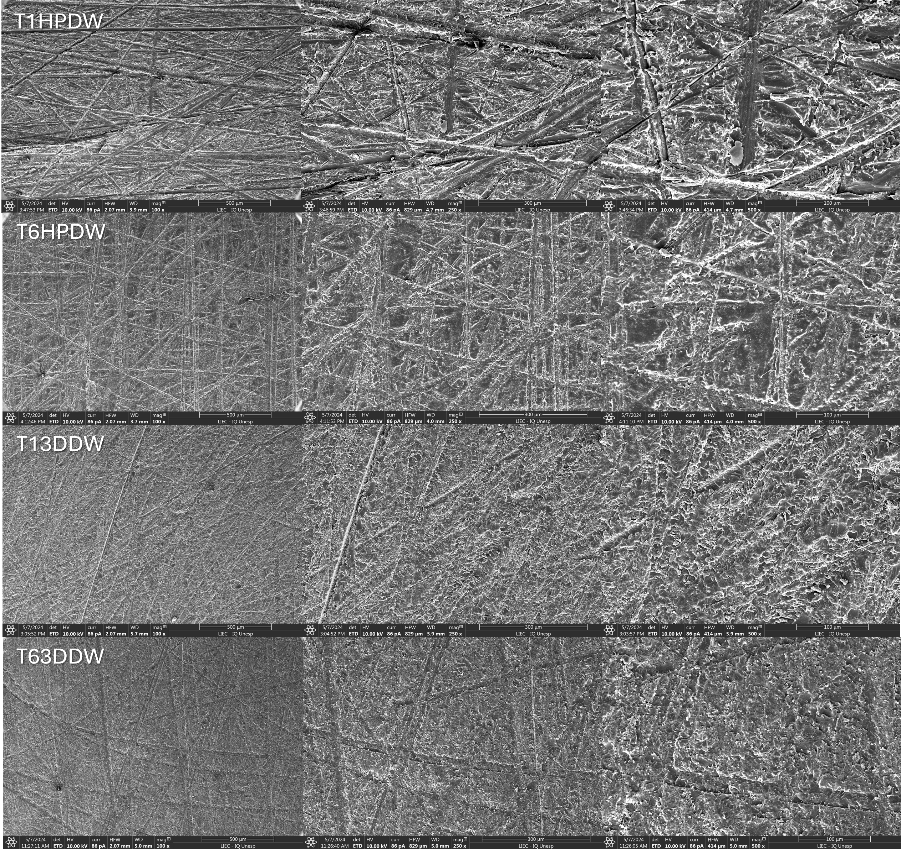


Fig. S10.3 SEM micrographs of sample surfaces immersed in sodium hypochlorite (SH) at T1 and T6 for both resin groups, conventional heat-polymerized (HP) and 3D-printed (3D), acquired at 100x, 250x and 500x, respectively. *Note*. T1 and T6 refers to specimens immersed in the solutions for 1 and 6 months.


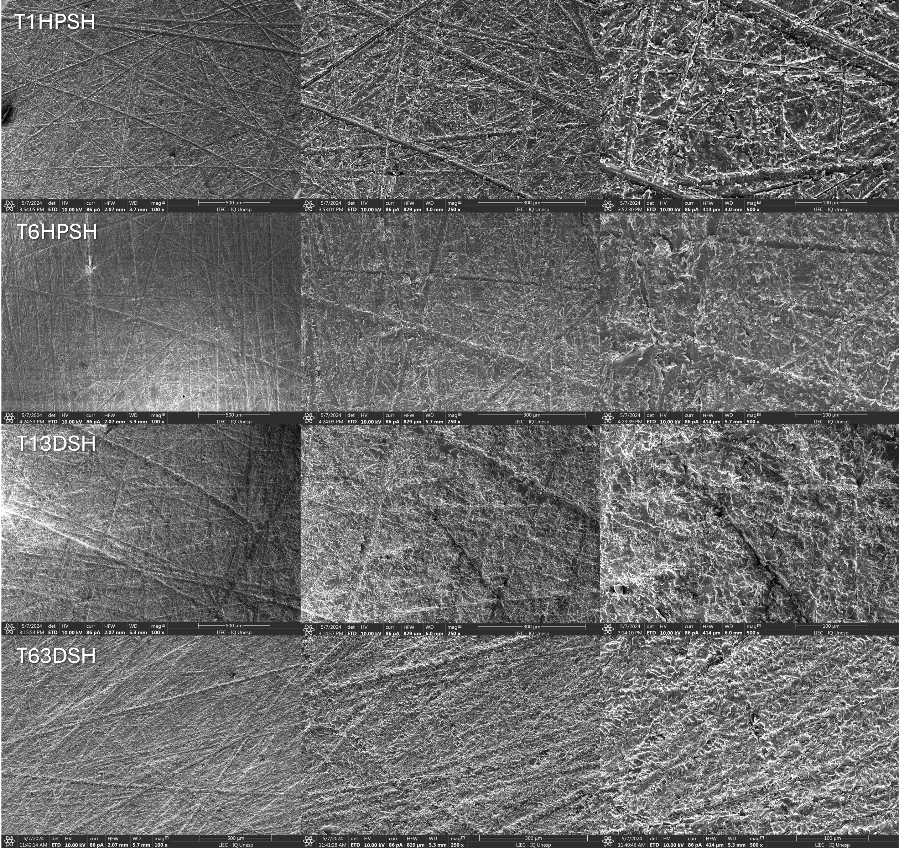


Fig. S10.4 SEM micrographs of sample surfaces immersed in chlorhexidine digluconate (CD) at T1 and T6 for both resin groups, conventional heat-polymerized (HP) and 3D-printed (3D), acquired at 100x, 250x and 500x, respectively. *Note*. T1 and T6 refers to specimens immersed in the solutions for 1 and 6 months.


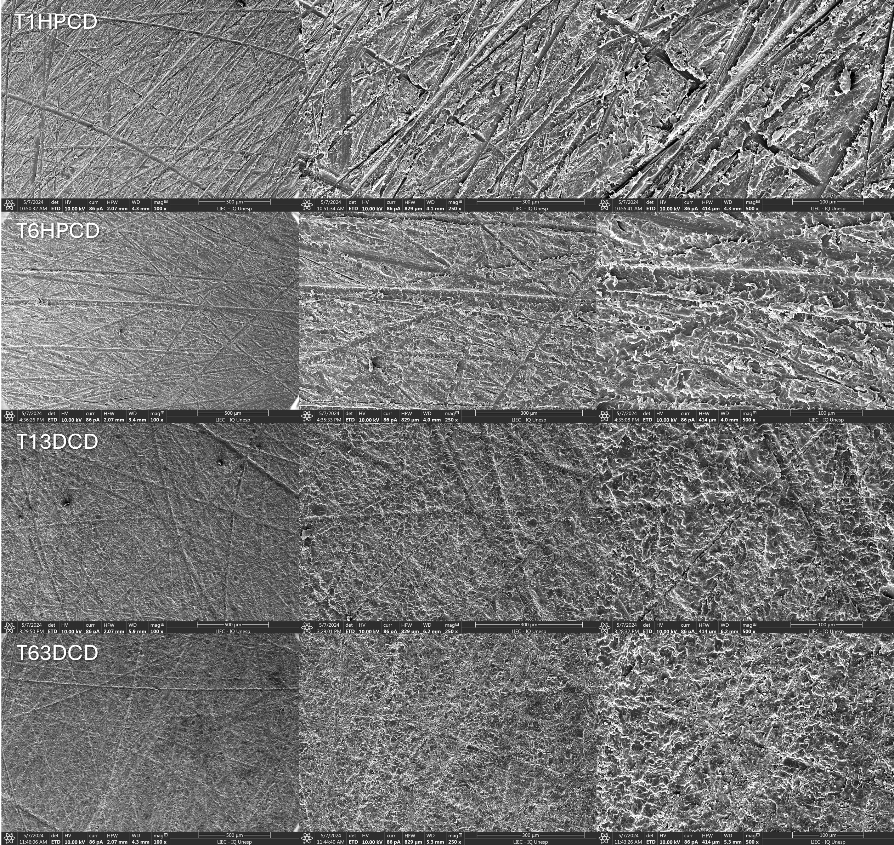


Fig. S10.5 SEM micrographs of sample surfaces immersed in Lifebuoy soap® (LS) at T1 and T6 for both resin groups, conventional heat-polymerized (HP) and 3D-printed (3D), acquired at 100x, 250x and 500x, respectively. *Note*. T1 and T6 refers to specimens immersed in the solutions for 1 and 6 months.


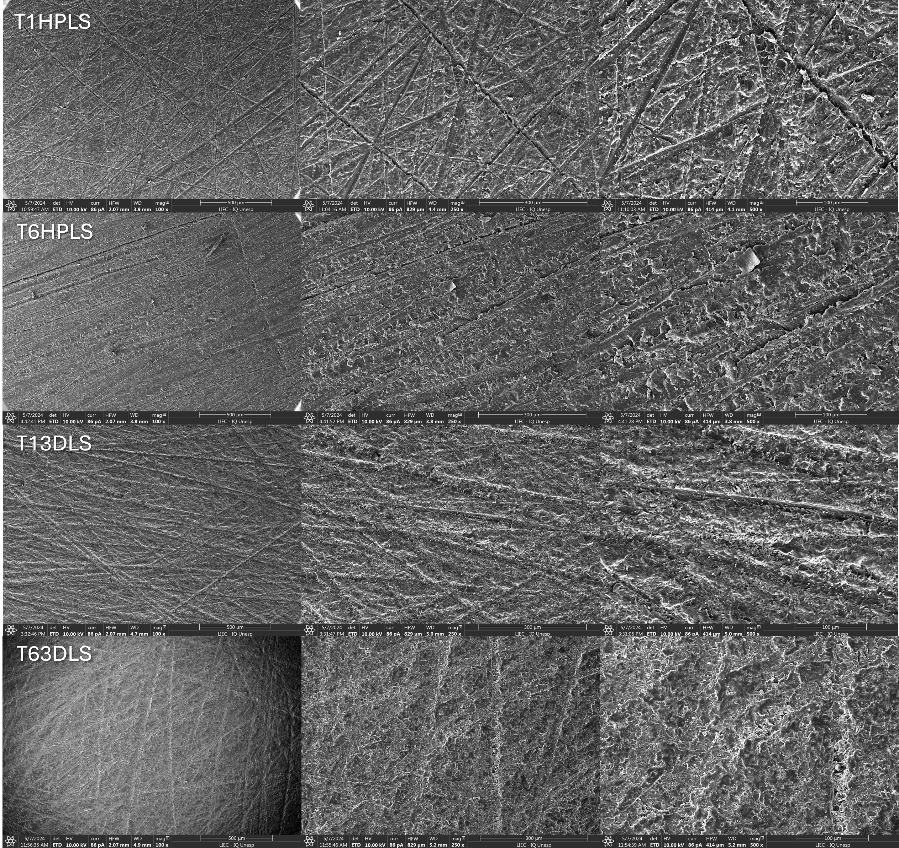


Table. S11 Three-way ANOVA for *C. albicans* proliferation (log10 CFU/mL). *Note*. Bold indicates statistically significant values (p < 0.05).

|  | | **Sum of Squares** | | **df** | | **Mean Square** | | **F** | | **p** |
| --- | --- | --- | --- | --- | --- | --- | --- | --- | --- | --- |
| Resin type | 12.45 | | 1 | | 12.455 | | 29.71 | | **< .001** | |
| Denture cleansers | 16.76 | | 3 | | 5.586 | | 13.33 | | **< .001** | |
| Immersion time | 13.44 | | 3 | | 4.480 | | 10.69 | | **< .001** | |
| Resin ✻ Cleansers | 11.06 | | 3 | | 3.687 | | 8.80 | | **< .001** | |
| Resin ✻ Time | 3.41 | | 3 | | 1.137 | | 2.71 | | **0.046** | |
| Cleansers ✻ Time | 36.31 | | 9 | | 4.034 | | 9.62 | | **< .001** | |
| Resin ✻ Cleansers ✻ Time | 19.82 | | 9 | | 2.203 | | 5.25 | | **< .001** | |
| Residual | 107.30 | | 256 | | 0.419 | |  | |  | |

Table. S12 Three-way ANOVA for *C. albicans* metabolism (RFU). *Note*. Bold indicates statistically significant values (p < 0.05).

|  | **Sum of Squares** | **df** | **Mean Square** | **F** | **p** |
| --- | --- | --- | --- | --- | --- |
| Resin type | 4.57e+7 | 1 | 4.57e+7 | 208.6 | **< .001** |
| Denture cleansers | 1.28e+8 | 3 | 4.28e+7 | 195.4 | **< .001** |
| Immersion time | 2.95e+8 | 3 | 9.82e+7 | 448.6 | **< .001** |
| Resin ✻ Cleansers | 8.56e+7 | 3 | 2.85e+7 | 130.2 | **< .001** |
| Resin ✻ Time | 1.79e+8 | 3 | 5.98e+7 | 272.9 | **< .001** |
| Cleansers ✻ Time | 1.68e+8 | 9 | 1.87e+7 | 85.4 | **< .001** |
| Resin ✻ Cleansers ✻ Time | 2.30e+8 | 9 | 2.56e+7 | 116.7 | **< .001** |
| Residual | 5.61e+7 | 256 | 218949 |  |  |

Fig. S13 Residual antimicrobial effect through *C. albicans* proliferation (Log 10 CFU/mL) and *C. albicans* biofilm metabolism (RFU) tests of the investigated groups of heat-polymerized (HP) and 3D-printed resins (3D) immersed in the solutions namely distilled water (DW), 1% sodium hypochlorite (SH), 2% chlorhexidine digluconate (CD) and soap solution (LS, Lifebuoy®), at zero or baseline (T0), one (T1), three (T3) and six (T6) months. (a) CFU assay methodology. (b) Log10 CFU/mL values according to the groups of resin (mean ± standard error of the mean). (c) Log10 CFU/mL values according to immersion times (mean ± standard deviation). (d) Log10 CFU/mL values according to interaction between solution and resin over time (mean ± standard deviation). (e) AlamarBlue assay methodology. (f) RFU values according to the groups of resin (mean ± standard error of the mean). (g) RFU values according to immersion times (mean ± standard deviation). (h) RFU values according to interaction between solution and resin over time (mean ± standard deviation). *Note.* Different lowercase letters above bars denote statistically significant differences between groups (Tukey’s test, p < 0.05). Asterisks above bars indicate significant differences (**** p < 0.0001) and “ns” denotes non-significant difference.


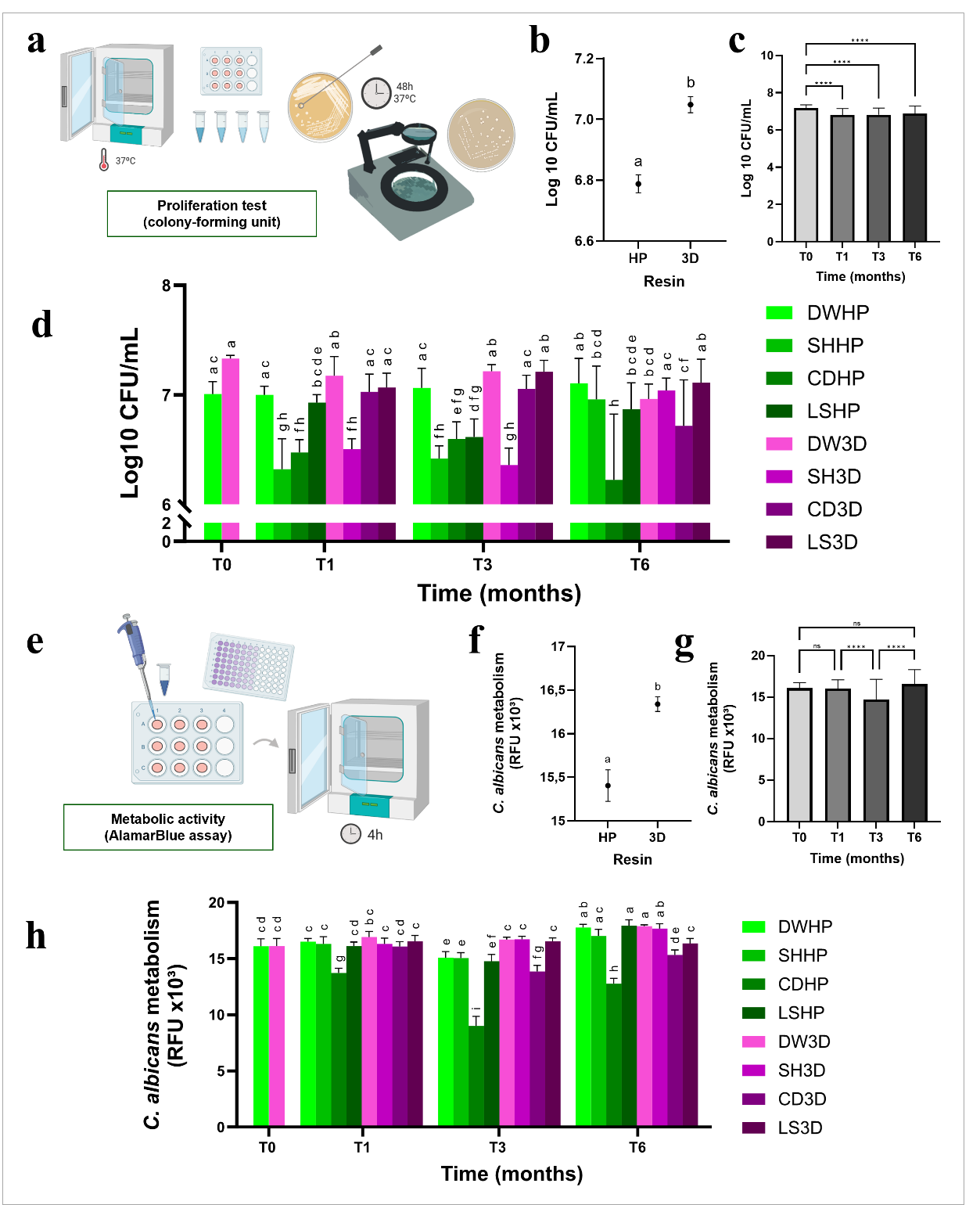


Table. S14 Mean ± standard deviation of cell metabolism (%) compared to control group according to resin type, disinfectant solution, and immersion time. *Note*. Superscript numbers indicate cytotoxicity classification of the groups according to ISO 10993-12:2007 and bold values represent slightly and moderately cytotoxic results.

| **Time** | | | | | |
| --- | --- | --- | --- | --- | --- |
| **Resin type** | **Solutions** | **T0** | **T1** | **T3** | **T6** |
| **Heat-polymerized resin (HP)** | DW | 97.6 ± 2.14^0^ | 95.7 ± 3.63^0^ | 95.6 ± 0.76^0^ | 100.0 ± 7.82^0^ |
|  | SH | 97.6 ± 2.14^0^ | 92.6 ± 3.66^0^ | 92.8 ± 2.23^0^ | 96.2 ± 5.70^0^ |
|  | CD | 97.6 ± 2.14^0^ | 88.2 ± 5.67^0^ | **45.2 ± 6.31^2^** | 70.8 ± 1.98^0^ |
|  | LS | 97.6 ± 2.14^0^ | 84.1 ± 4.78^0^ | 85.1 ± 1.79^0^ | 94.1 ± 6.26^0^ |
| **3D-printed resin (3D)** | DW | 98.8 ± 1.38^0^ | 97.6 ± 0.00^0^ | 95.4 ± 2.52^0^ | 101.5 ± 2.35^0^ |
|  | SH | 98.8 ± 1.38^0^ | 83.9 ± 5.10^0^ | 84.5 ± 1.18^0^ | 98.6 ± 4.77^0^ |
|  | CD | 98.8 ± 1.38^0^ | **64.6 ± 5.25^1^** | **55.0 ± 9.55^1^** | 87.9 ± 5.49^0^ |
|  | LS | 98.8 ± 1.38^0^ | 84.0 ± 8.71^0^ | 84.7 ± 1.95^0^ | 94.7 ± 5.00^0^ |

Table. S15 Three-way ANOVA for Cell metabolism (%). Note. Bold indicates statistically significant values (p < 0.05).

|  | **Sum of Squares** | **df** | **Mean Square** | **F** | **p** |
| --- | --- | --- | --- | --- | --- |
| Resin type | 3.25 | 1 | 3.25 | 0.170 | 0.680 |
| Denture cleansers | 19030.80 | 3 | 6343.60 | 333.018 | **< .001** |
| Immersion time | 13808.33 | 3 | 4602.78 | 241.630 | **< .001** |
| Resin ✻ Cleansers | 248.12 | 3 | 82.71 | 4.342 | **0.005** |
| Resin ✻ Time | 1592.95 | 3 | 530.98 | 27.875 | **< .001** |
| Cleansers ✻ Time | 11838.55 | 9 | 1315.39 | 69.054 | **< .001** |
| Resin ✻ Cleansers ✻ Time | 3152.47 | 9 | 350.27 | 18.388 | **< .001** |
| Residual | 4876.50 | 256 | 19.05 |  |  |

Table. S16 Three-way ANOVA for Cell metabolism (RFU). *Note*. Bold indicates statistically significant values (p < 0.05).

|  | **Sum of Squares** | **df** | **Mean Square** | **F** | **p** |
| --- | --- | --- | --- | --- | --- |
| Resin type | 1.11e+7 | 1 | 1.11e+7 | 259.38 | **< .001** |
| Denture cleansers | 3.37e+7 | 3 | 1.12e+7 | 263.20 | **< .001** |
| Immersion time | 7.37e+8 | 3 | 2.46e+8 | 5752.95 | **< .001** |
| Resin ✻ Cleansers | 645554 | 3 | 215185 | 5.04 | **0.002** |
| Resin ✻ Time | 1.88e+7 | 3 | 6.28e+6 | 147.06 | **< .001** |
| Cleansers ✻ Time | 1.96e+7 | 9 | 2.18e+6 | 51.09 | **< .001** |
| Resin ✻ Cleansers ✻ Time | 4.62e+6 | 9 | 513019 | 12.02 | **< .001** |
| Residual | 1.09e+7 | 256 | 42693 |  |  |

Fig. S17 Residual cytotoxicity effect through L-929 cell metabolism (RFU) test of the investigated groups of heat-polymerized (HP) and 3D-printed resins (3D) immersed in the solutions namely distilled water (DW), 1% sodium hypochlorite (SH), 2% chlorhexidine digluconate (CD) and soap solution (LS, Lifebuoy®), at zero or baseline (T0), one (T1), three (T3) and six (T6) months. (a) AlamarBlue assay methodology. (b) % Cell metabolism values according to the groups of resin (mean ± standard error of the mean). (c) % Cell metabolism values according to immersion times (mean ± standard deviation). (d) % Cell metabolism values according to interaction between solution and resin over time (mean ± standard deviation). *Note.* The dashed line marks the 70% ISO threshold for non-cytotoxicity. (e) RFU values according to the groups of resin (mean ± standard error of the mean). (f) RFU values according to immersion times (mean ± standard deviation). (g) RFU values according to interaction between solution and resin over time (mean ± standard deviation). *Note.* Different lowercase letters above bars denote statistically significant differences between groups (Tukey’s test, p < 0.05). Asterisks above bars indicate significant differences (* p ≤ 0.0332, ** p ≤ 0.0021, **** p < 0.0001), and “ns” denotes non-significant difference.


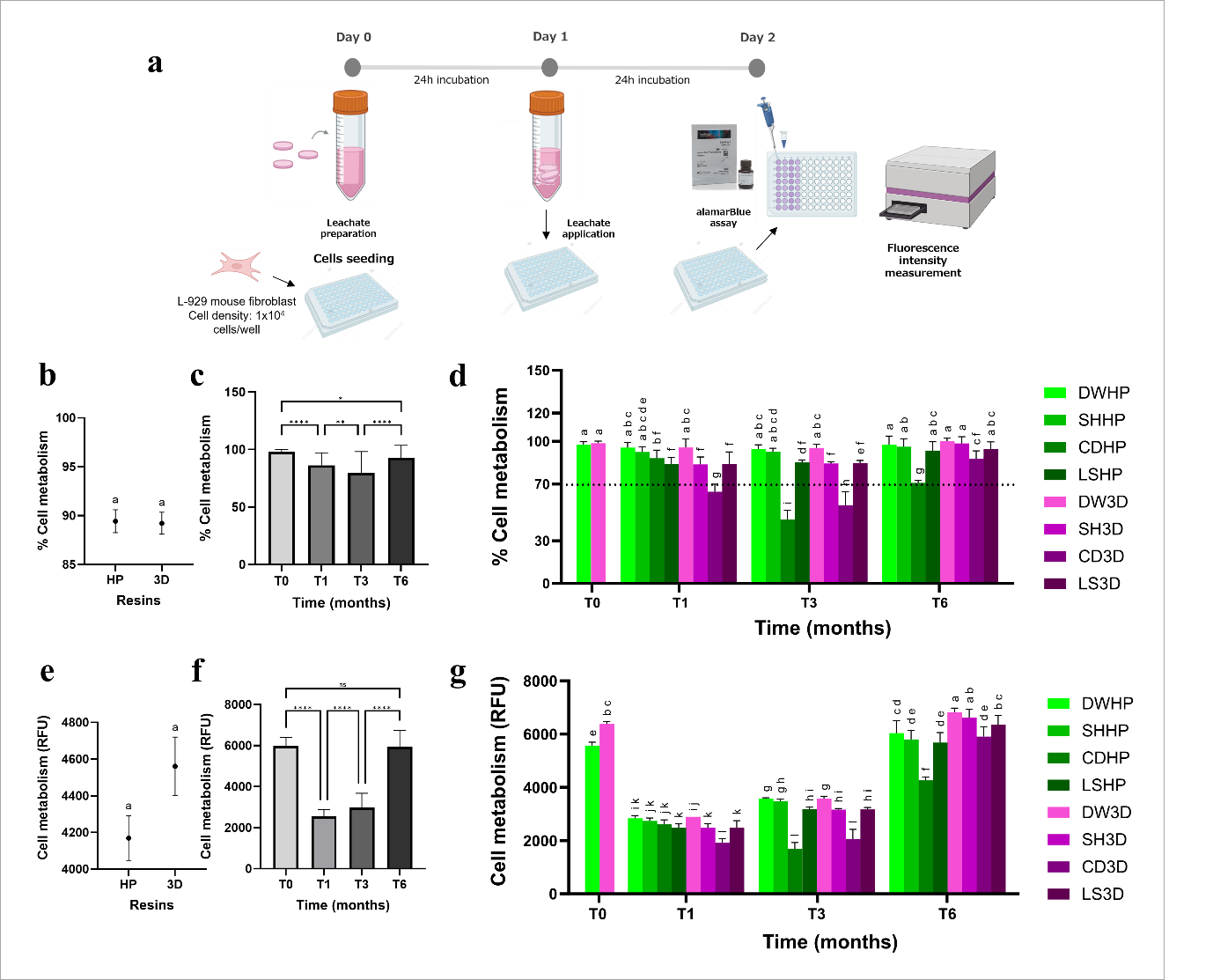

Supplement: Supplementary file 1 — Supplementary information [file 10856_2026_7049_MOESM1_ESM.docx]
